# Nanoparticle-Enabled Modulation of the Bone Immune Microenvironment for Enhanced Regeneration

**DOI:** 10.3390/bioengineering13070755

**Published:** 2026-06-27

**Authors:** Güleycan Dedecengiz Varol, Fatih Ciftci, Ali Can Özarslan, Azime Erarslan, Ahmet Akif Kızılkurtlu

**Affiliations:** 1 Faculty of Chemistry and Metallurgy, Department of Bioengineering, Yıldız Technical University, Istanbul 34220, Turkey; guleycan1997@gmail.com (G.D.V.); azime@yildiz.edu.tr (A.E.); 2 Faculty of Electrics and Electronics, Department of Biomedical Engineering, Yıldız Technical University, Istanbul 34220, Turkey; 3 Faculty of Engineering, Department of Biomedical Engineering, Fatih Sultan Mehmet Vakıf University, Istanbul 34015, Turkey; fciftci@fsm.edu.tr; 4 BioriginAI Research Group, Department of Biomedical Engineering, Fatih Sultan Mehmet Vakıf University, Istanbul 34015, Turkey; 5 Biomedical Electronic Design Application and Research Center (BETAM), Fatih Sultan Mehmet Vakıf University, Istanbul 34015, Turkey; 6 Faculty of Engineering, Department of Metallurgy and Materials Engineering, Istanbul University-Cerrahpaşa, Istanbul 34320, Turkey; ali.ozarslan@iuc.edu.tr; 7 Faculty of Engineering and Natural Sciences, Department of Biomedical Engineering, Atlas University, Istanbul 34408, Turkey

**Keywords:** osteoimmunomodulation, bone regeneration, nanoparticles, macrophage polarization, immune microenvironment

## Abstract

Bone regeneration is governed by a tightly coordinated interplay between skeletal cells, immune cells, vascular components, and signaling networks within a dynamic microenvironment. Increasing evidence from osteoimmunology demonstrates that immune regulation is not merely supportive but mechanistically determinative of regenerative outcomes. Dysregulated or persistent inflammation can impair osteogenesis, whereas timely immune resolution promotes angiogenesis and matrix deposition. In this context, nanotechnology has enabled the development of nanoparticles (NPs) that function not only as delivery vehicles but also as active modulators of the bone immune microenvironment. Immunomodulatory NPs can be engineered to deliver bioactive agents, regulate cytokine networks, and influence immune cell phenotypes, particularly macrophage polarization, at defined stages of healing. Through tailored surface chemistry, targeting ligands, and stimuli-responsive release mechanisms, NPs can achieve spatially localized and temporally controlled modulation of inflammatory and reparative phases, thereby enhancing osteogenesis and vascular integration. This review provides a comprehensive overview of organic, inorganic, and hybrid NP platforms applied to bone regeneration, with emphasis on their mechanisms of immune modulation, strategies for cell-specific targeting, and approaches for sequential regulation of inflammatory resolution and tissue repair. By integrating advances in materials science and immunology, NP-enabled platforms have the potential to transform bone regeneration from passive structural repair into precision immune-guided healing.

## 1. Introduction

Bone possesses a remarkable intrinsic capacity for self-repair; however, this regenerative potential becomes compromised in large defects, traumatic injuries, infections, metabolic disorders, and aging-associated conditions [1,2]. In such cases, impaired vascularization, dysregulated inflammation, and insufficient osteogenic signaling collectively hinder complete structural and functional restoration. Conventional bone grafting approaches, including autografts, allografts, and synthetic substitutes, primarily provide structural support or osteoconductive matrices, yet they often fail to address the complex immunological dimension of bone healing. Increasing evidence now establishes that successful bone regeneration is not governed solely by osteoblast lineage activity but is critically dependent on tightly coordinated immune responses within the bone microenvironment. The concept of osteoimmunology has emerged to describe the reciprocal interactions between immune cells and skeletal cells during bone remodeling and repair. Immediately following injury, immune cells infiltrate the defect site and initiate an inflammatory cascade essential for debris clearance and early regenerative signaling [3,4]. However, the magnitude, duration, and resolution of this immune response determine whether healing progresses toward regeneration or chronic inflammation and fibrotic encapsulation. Macrophages, dendritic cells, and T lymphocytes dynamically regulate cytokine networks, angiogenic processes, and osteogenic differentiation. A timely transition from pro-inflammatory to pro-regenerative immune phenotypes is therefore indispensable for effective bone regeneration [5,6,7]. Failure to achieve this balance contributes to delayed union, non-union, and pathological bone loss. Recent advances in nanotechnology have introduced new opportunities to actively modulate the immune microenvironment during bone regeneration. Unlike traditional biomaterials that passively integrate into host tissue, NPs can be engineered to function as dynamic regulators of immune signaling. Their nanoscale dimensions enable preferential interaction with immune cells, particularly macrophages, while their tunable surface chemistry, degradation behavior, and cargo-loading capacity allow precise control over bioactive cue presentation [8,9]. NPs may act as carriers for osteoinductive factors, anti-inflammatory agents, nucleic acids, or immunoregulatory cytokines [10,11,12]. Recent research has shown that NP-based systems can also actively regulate macrophage polarization and reshape the bone immune microenvironment, thereby supporting both osteogenesis and bone regeneration [13]. Among osteoinductive molecules, bone morphogenetic proteins (BMPs), particularly BMP-2 and BMP-7, have attracted considerable attention because of their ability to stimulate osteogenic differentiation and enhance bone regeneration in challenging clinical conditions. Recent clinical evidence further highlights the therapeutic potential of BMP-based approaches for managing complex bone defects and medication-related osteonecrosis of the jaw, although concerns regarding dosage optimization, safety, and localized delivery remain important considerations [14].

Importantly, beyond delivery functions, their intrinsic physicochemical properties such as ion release, surface topography, redox activity, and protein corona formation can directly influence immune cell behavior. Inorganic NPs, including calcium phosphate, bioactive glass, and metal oxide systems, can mimic the mineral phase of native bone while releasing bioactive ions that regulate both osteogenic and inflammatory pathways. Organic polymer-based NPs provide controlled and sustained delivery of therapeutic agents, enabling spatiotemporal tuning of immune modulation. Hybrid nanostructures integrate these complementary advantages to create multifunctional platforms capable of synchronizing immune resolution with osteogenesis and angiogenesis. Such osteoimmunomodulatory NPs represent a paradigm shift from scaffold-centered design toward immune-informed regenerative engineering [15,16,17]. Although NP-based bone regeneration has been widely reviewed in terms of osteogenesis, angiogenesis, drug delivery, and material bioactivity, most previous studies have considered these processes as separate therapeutic targets rather than as interconnected outcomes of immune regulation [18,19]. Existing reviews have primarily focused on the osteogenic performance, drug delivery capabilities, angiogenic potential, or material characteristics of nanoparticle-based systems for bone regeneration. Although these studies have substantially advanced the field, immune regulation has generally been considered a secondary outcome rather than a central determinant of regenerative success. Consequently, the dynamic interplay among nanoparticle physicochemical properties, immune cell behavior, inflammatory resolution, and tissue regeneration remains insufficiently integrated within the current literature. In particular, limited attention has been devoted to understanding how nanoparticle design parameters, including surface chemistry, ion release profiles, targeting ligands, and stimuli-responsive functionalities, influence the spatiotemporal regulation of the osteoimmune microenvironment throughout the different phases of bone healing. Moreover, translational considerations, such as long-term biosafety, manufacturing reproducibility, regulatory challenges, and clinical applicability, are often discussed only briefly. To address these knowledge gaps, the present review adopts a mechanism-oriented perspective on nanoparticle-enabled osteoimmunomodulation. Rather than categorizing nanoplatforms solely according to their material composition or cargo delivery functions, this review critically evaluates how organic, inorganic, and hybrid nanoparticles actively regulate immune cell phenotypes and coordinate inflammatory resolution, angiogenesis, and osteogenesis. Furthermore, recent advances are discussed within a translational framework that highlights current limitations, emerging design principles, and future directions for clinically relevant immune-guided bone regeneration. Recent evidence indicates that macrophage polarization and the local osteoimmune microenvironment are not merely supportive factors but central regulators that coordinate inflammatory resolution, vascularization, osteogenic differentiation, and bone remodeling [18]. Therefore, the novelty of this review lies in its specific focus on NP-enabled osteoimmunomodulation as an integrated regulatory strategy, rather than on NPs only as passive carriers, osteoconductive additives, or conventional drug-delivery systems. Unlike previous reviews that mainly classify nanomaterials according to composition or regenerative function, this review evaluates how organic, inorganic, and hybrid NPs regulate immune cell behavior through ion release, surface chemistry, redox activity, targeting ligands, and stimuli-responsive release mechanisms. In this context, the review provides a mechanism-oriented framework linking NP physicochemical properties with macrophage phenotype switching, cytokine regulation, angiogenic–osteogenic coupling, and translational limitations. This perspective clarifies the distinctive contribution of the present review and positions NP-based osteoimmunomodulation as a rational design principle for next-generation immune-guided bone regenerative therapies.

Targeting specificity and temporal control further enhance the therapeutic potential of NP systems. By functionalizing NPs with bone-homing ligands or immune-cell-specific moieties, localized modulation of inflammatory processes can be achieved while minimizing systemic immune suppression. Stimuli-responsive NPs capable of responding to pH shifts, oxidative stress, or enzymatic activity provide adaptive release profiles aligned with the evolving phases of bone healing (Table 1). For example, bone-targeted lipid and lipid–polymer hybrid NPs have recently been used to improve the local delivery of osteogenic or gene-regulatory cargos, supporting more selective and sustained bone regenerative responses [20,21]. These innovations collectively enable NPs to coordinate early inflammatory regulation with later-stage tissue formation, thereby improving regenerative efficiency. Despite these advances, translation of NP-enabled osteoimmunomodulation into clinical practice requires comprehensive evaluation of safety, reproducibility, long-term biodistribution, and immune specificity. Differences between animal models and human immune responses, manufacturing scalability challenges, and regulatory complexity remain critical considerations. A deeper mechanistic understanding of immune–bone coupling will be essential to guide the rational design of next-generation NP systems. This review provides a comprehensive examination of NP-based strategies for modulating the bone immune microenvironment to enhance bone regeneration. We discuss material classifications, surface functionalization principles, mechanisms of immune regulation, targeted delivery approaches, and temporal control strategies. Furthermore, we address translational limitations and future perspectives that may guide the development of clinically viable osteoimmunomodulatory nanotherapies. By integrating insights from immunology, materials science, and tissue engineering, NP-enabled platforms hold the potential to transform bone regeneration from passive structural repair toward intelligent immune-guided healing.

**Table 1:** Representative NP platforms enabling osteoimmunomodulation for bone regeneration.

Platform (NP + Carrier/Scaffold)	NP Class	Immunomodulatory Action (In Vivo/In Situ)	Regeneration-Relevant Outcome	Reference
Bioactive glass/sodium alginate injectable hydrogel	Inorganic (bioactive glass)	Promoted macrophage shift toward pro- regenerative phenotypes (immune tuning via ionic cues)	Improved regenerative microenvironment and pro-repair tissue responses	[22]
Tetra-PEG (PEG)/nHAp/chitosan composite hydrogel (±PTH depot)	Hybrid (polymer + mineral NP)	Induced M2-skewed macrophage polarization and dampened M1-associated signaling (reported via TLR4/NF-κB axis)	Enhanced osteogenesis and improved healing in osteoporotic defect model	[23]
Mesoporous bioactive glass (MBG)/protein-integrated bone graft	Inorganic (MBG NP)	Programmed local immune milieu through material/ionic interactions (macrophage-oriented osteoimmune tuning)	Promoted bone regeneration via immune–osteogenic coupling	[24]
Se NP-decorated MgFe-LDH nanosheets on bioactive glass scaffold	Hybrid (nano-Se + LDH on scaffold)	Reactive Oxygen Species (ROS) /inflammation regulation to bias innate immune response toward regenerative profiles	Improved bone regeneration performance with immune-compatible healing	[25]
Nano-MgO-loaded thermosensitive HPCH@HA hydrogel	Inorganic NP in hydrogel	Drove macrophages toward M2-like behavior; supported T-cell activation and anti-inflammatory milieu	Accelerated repair in cranial defect model; enhanced angiogenesis/osteogenesis	[5]
Dynamic nanoclay-coassembled adhesive hydrogel (polyphenol/polypeptide/clay nanosheets)	Hybrid (nanoclay + polymeric network)	Proactive immunomodulation with antioxidant/anti-inflammatory behavior at defect site	Improved defect adaptation + accelerated bone defect healing	[26]
3D-printed GelMA/nHAp/melanin NP composite scaffold	Hybrid (GelMA + mineral + melanin NP)	Promoted M2 polarization (reported via Lif-associated signaling)	Enhanced cranial defect healing in vivo; improved bone formation	[27]
GelMA/LPS + PLLA/OPC dual immune-modulatory composite scaffold	Hybrid composite scaffold	Orchestrated immune response (macrophage recruitment/phenotype shaping) in a stage-aware manner	Enhanced bone regeneration via synchronized immune regulation	[28]
Thermosensitive antibacterial nanocomposite hydrogel (periodontitis bone regeneration)	Hybrid nanocomposite	Guided macrophage polarization while controlling inflammation in immune-active defects	Improved bone regeneration under inflammatory/infectious conditions	[29]

## 2. Literature Search Strategy

This narrative review was prepared following the general principles outlined in the Preferred Reporting Items for Systematic Reviews and Meta-Analyses (PRISMA) 2020 statement to improve transparency and reproducibility in the identification, selection, and synthesis of the relevant literature. Although a formal systematic review or meta-analysis was not conducted, key elements of the PRISMA framework were incorporated to ensure a structured and comprehensive review process.

A comprehensive literature search was performed using the PubMed, Web of Science, Scopus, and Google Scholar databases to identify studies relevant to NP-mediated osteoimmunomodulation and bone regeneration. The search covered publications from January 2020 to March 2026 and was limited to articles published in English.

The search strategy combined Medical Subject Headings (MeSH) and free-text keywords related to bone regeneration, osteoimmunology, and nanotechnology. Representative search terms included “bone regeneration”, “osteoimmunology”, “bone immune microenvironment”, “nanoparticles”, “nanomedicine”, “immune modulation”, “macrophage polarization”, “bone tissue engineering”, “targeted delivery”, and “stimuli-responsive nanoparticles”.

Original research articles, translational studies, preclinical investigations, and high-quality review articles directly addressing nanoparticle-mediated modulation of the bone immune microenvironment were considered eligible for inclusion. Studies focusing exclusively on conventional bone graft materials without immunomodulatory mechanisms, non-bone tissue applications, conference abstracts, editorials, patents, book chapters, and articles lacking sufficient mechanistic or experimental detail were excluded.

Full-text articles were subsequently evaluated according to predefined inclusion and exclusion criteria. Additional studies were identified through manual screening of the reference lists of eligible publications to ensure comprehensive coverage of the field.

## 3. NPs in Bone Regeneration

**Figure 1:**
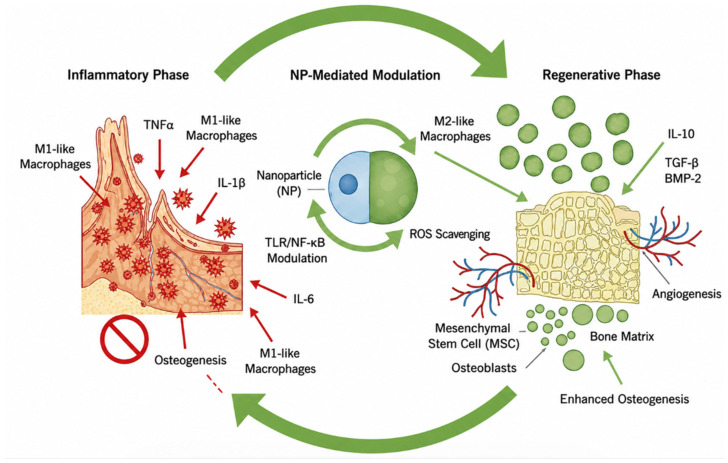
The schematic illustrates the dynamic transition of the bone defect site from an acute inflammatory state to a pro-regenerative niche, where the early microenvironment is dominated by pro-inflammatory M1-like macrophages secreting cytokines such as TNF-α, IL-6, and IL-1β, which can impair spontaneous osteogenesis if their expression persists. Engineered NPs act as active regulators to modulate these immune responses, interacting with infiltrating cells to suppress pro-inflammatory signaling and facilitate ROS scavenging, thereby driving a phenotypic shift toward reparative M2-like macrophages. This normalized osteoimmune milieu promotes the secretion of pro-regenerative factors like IL-10, TGF-β, and BMP-2, which collectively recruit Mesenchymal Stem Cells (MSCs) and coordinate synchronized angiogenesis and osteogenesis to achieve enhanced bone matrix deposition and structural repair. Green arrows indicate regenerative processes and phenotypic transitions, whereas red arrows represent inflammatory signaling and inhibitory effects. This figure was created in BioRender and further refined using Adobe Illustrator 2024. Created in BioRender. Dedecengiz Varol, G. (2026).

Bone regeneration is a highly orchestrated biological process involving the interplay between osteogenic cells, immune cells, vascular components, and the extracellular matrix. In recent years, NPs have emerged as powerful tools in bone tissue engineering due to their tunable physicochemical properties, high surface area, and ability to interact dynamically with the bone immune microenvironment. By mimicking native bone mineral, delivering bioactive cues, or modulating inflammatory signaling, NPs can actively regulate cellular behavior and promote functional bone regeneration. This section provides an overview of NP types commonly employed in bone regeneration and discusses how their material composition and surface characteristics influence biological performance. Successful bone regeneration is fundamentally determined by the temporal coordination of the immune response within the local microenvironment. As illustrated in Figure 1, the immediate aftermath of injury is characterized by an inflammatory phase dominated by M1-like macrophages. These cells secrete pro-inflammatory cytokines such as TNF-α, IL-6, and IL-1β, which, if persistent, impair the osteogenic differentiation of mesenchymal stem cells (MSCs).

NP-enabled platforms act as active regulators to modulate this osteoimmune niche. As shown in the NP-mediated modulation phase of Figure 1, engineered NPs interact with infiltrating immune cells to suppress pro-inflammatory signaling (NF-κB pathways) and facilitate ROS scavenging. This intervention drives the essential phenotypic shift from M1-like toward reparative M2-like macrophages.

The resulting regenerative phase establishes a normalized osteoimmune milieu where M2 macrophages secrete pro-regenerative factors such as IL-10, TGF-β, and BMP-2. These signals resolve local inflammation while actively recruiting MSCs and coordinating synchronized angiogenesis and osteogenesis. By guiding this transition, NP-based strategies transform bone regeneration from passive structural support into precision immune-guided healing.

### 3.1. Types and Materials

NPs employed in bone regeneration can be broadly classified into organic, inorganic, and hybrid systems based on their material composition [30,31]. This classification is particularly relevant in the context of osteoimmunomodulation, as each material class interacts with the bone immune microenvironment through distinct physicochemical and biological mechanisms. While inorganic NPs often provide structural and osteoconductive cues, organic NPs excel in controlled delivery and immune regulation [18,32,33,34]. Hybrid NPs aim to integrate these complementary features into multifunctional platforms capable of synchronizing immune modulation with osteogenesis [35,36].

**Figure 2:**
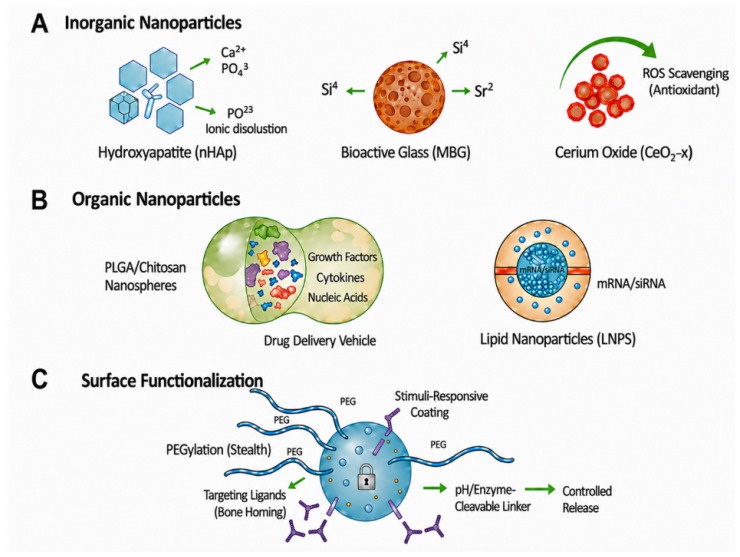
Schematic overview of nanoparticulate platforms for targeted modulation of the osteoimmune microenvironment. (**A**) Representative inorganic NPs, including hydroxyapatite (nHAp), mesoporous bioactive glass (MBG), and CeO_2_ − x, highlighting their ion release profiles and ROS-scavenging properties. (**B**) Representative organic NP systems, including PLGA/chitosan nanospheres and lipid NPs (LNPs), designed for the delivery of growth factors, cytokines, nucleic acids, mRNA, and siRNA. (**C**) Advanced surface functionalization strategies, including PEGylation, targeting ligands for bone homing, stimuli-responsive coatings, and pH/enzyme-cleavable linkers for controlled and site-specific therapeutic release. The figure was initially designed using BioRender and subsequently edited and finalized with Adobe Illustrator 2024. Dedecengiz Varol, G. (2026).

The rational design of NPs for bone regeneration integrates material composition with precise surface chemistry. As shown in Figure 2, inorganic NPs such as hydroxyapatite (nHAp) and mesoporous bioactive glass (MBG) are fundamental to osteoimmunomodulation through the release of bioactive ions like Ca^2+^, Si^4+^, and Sr^2+^. These dissolution products directly influence macrophage polarization toward a pro-regenerative phenotype. Furthermore, cerium oxide (CeO_2_) NPs contribute potent antioxidant properties by scavenging ROS to mitigate oxidative stress at the defect site.

Organic NP systems, including Poly(lactic-co-glycolic acid) (PLGA) and chitosan nanospheres, serve as versatile delivery vehicles for growth factors, cytokines, and nucleic acids (mRNA/siRNA). Surface engineering strategies, such as Polyethylene glycol (PEG) modification (PEGylation), provide an immune “stealth” effect to minimize non-specific protein adsorption and systemic clearance. Additionally, the conjugation of bone-homing ligands, such as Alendronate or Asp8 peptides, ensures the selective accumulation of nanocarriers within mineralized niches. By incorporating stimuli-responsive coatings with pH-sensitive or enzyme-cleavable linkers, these platforms achieve precise spatiotemporal control, releasing therapeutic payloads in response to specific triggers like the acidic pH of an inflammatory defect.

#### 3.1.1. Inorganic NPs

Inorganic NPs constitute one of the most widely investigated material classes for bone regeneration due to their close resemblance to the mineral phase of native bone.

##### Calcium Phosphate-Based NPs

Calcium phosphate-based NPs, such as hydroxyapatite, tricalcium phosphate, and amorphous calcium phosphate, are particularly prominent owing to their excellent biocompatibility, bioactivity, and osteoconductivity [37,38,39,40,41,42]. At the nanoscale, these materials exhibit increased surface area and enhanced ion exchange capacity, which facilitate protein adsorption and promote osteogenic cell adhesion and differentiation. Beyond their direct effects on osteoblast lineage cells, calcium phosphate NPs play a crucial role in modulating the immune response during bone healing [43,44]. The controlled release of calcium and phosphate ions has been shown to influence macrophage behavior, favoring a transition from a pro-inflammatory phenotype toward a reparative, pro-osteogenic state [45,46]. This immunomodulatory capacity is especially relevant in the early inflammatory phase of bone regeneration, where excessive or prolonged inflammation can impair healing outcomes.

##### Bioactive Glass NPs

Bioactive glass NPs represent another important category of inorganic materials. Composed primarily of silica-based networks with calcium and phosphate components, these NPs release biologically active ions that stimulate angiogenesis and osteogenesis [47,48]. Ionic dissolution products, particularly silicon ions, have been reported to regulate macrophage polarization and reduce inflammatory cytokine secretion, thereby creating a microenvironment conducive to tissue regeneration [49,50]. The ability of bioactive glass NPs to simultaneously support vascularization and immune modulation makes them attractive candidates for complex bone defect repair. Consistently, selenium-doped mesoporous bioactive glass has been reported to regulate macrophage metabolism and polarization through ROS scavenging, resulting in improved bone regeneration in vivo [51]. Tian et al. investigated mesoporous bioactive glass NPs (MBGNs) functionalized with copper ions to enhance angiogenesis. They hypothesized that controlled release of Cu^2+^ and Si^4+^ from BGNs could upregulate pro-angiogenic factors such as VEGF and HIF-1α in endothelial cells. Their in vitro and in vivo results showed significantly increased capillary formation and enhanced bone regeneration in a rat calvarial defect model compared to non-functionalized controls, suggesting that bioactive glass ion dissolution can directly stimulate vascular ingrowth alongside osteogenesis [47]. In a separate approach, Zheng et al. examined the immunomodulatory properties of silicate glass NPs in the context of macrophage polarization. Their study was motivated by the recognition that excessive inflammatory responses retard healing, whereas early M2-like macrophage polarization supports tissue repair and vascular formation. They reported that ionic products from the glass nanomaterials suppressed pro-inflammatory cytokine production (TNF-α, IL-6) while enhancing expression of anti-inflammatory markers (IL-10, Arg-1) in murine macrophages. When implanted into critical-sized defects, treated defects exhibited improved vascular density and bone formation relative to controls, indicating a direct link between BGN-mediated immune modulation and subsequent regenerative outcomes [52]. Huang et al. focused on the dual action of Si-rich dissolution products on endothelial and immune cells. They reasoned that, beyond traditional osteogenic stimulation, silicon ions released from BGNs could act on diverse cell types in the defect microenvironment. Their mechanistic experiments revealed that Si-ion exposure increased nitric oxide production and angiogenic gene expression in human umbilical vein endothelial cells (HUVECs), while concurrently attenuating NF-κB signaling in macrophages, leading to reduced secretion of pro-inflammatory mediators. In a murine segmental bone defect model, these combined effects correlated with accelerated neovascularization and improved structural repair [50].

##### Metal and Metal Oxide NPs

Metal and metal oxide NPs, including titanium dioxide, iron oxide, zinc oxide, and CeO_2_, have also been explored for bone regenerative applications [53,54,55,56]. Iron oxide NPs are notable for their magnetic properties, which allow external manipulation and targeted delivery, while zinc- and cerium-based NPs exhibit antioxidant and anti-inflammatory activities. These properties are particularly valuable in mitigating oxidative stress and chronic inflammation, which are common features of impaired bone healing. Nevertheless, careful optimization of particle size, concentration, and surface chemistry is essential to balance bioactivity with biosafety. In a study by Kong et al., titanium surfaces were modified with TiO_2_ nanotubes produced by anodic oxidation to investigate their effect on bone regeneration mechanisms. They seeded bone marrow-derived mesenchymal stem cells (BMSCs) on surfaces with and without TiO_2_ nanotube topography and evaluated osteogenic differentiation in vitro and bone formation in vivo using a rat bone defect model. Kong et al. found that the nanotubular surfaces significantly enhanced key osteogenic markers, including ALP, Runx2, and osteocalcin, compared to smooth titanium, demonstrating that nanoscale TiO_2_ structures can stimulate osteogenesis. Mechanistically, they showed increased nuclear localization of the mechanosensitive protein Yap and elevated downstream Piezo1 expression, linking physical nanotopography to biological signaling pathways that promote bone formation [57]. Lee et al. evaluated TiO_2_ nanotube arrays on dental implants as a coating and delivery platform for recombinant human BMP-2, a potent osteogenic growth factor. They compared conventional implants with TiO_2_ nanotube-coated implants, and nanotube implants loaded with BMP-2, in an animal model. Their results showed that TiO_2_ nanotube surfaces alone increased bone-to-implant contact and bone remodeling, while BMP-2-loaded nanotubes produced the highest levels of new bone formation and remodeling around the implant threads. This suggests that TiO_2_ nanotube arrays can act both as osteoconductive surfaces and as drug reservoirs to enhance bone regeneration [58]. Wang et al. showed the mechanotransduction effects of TiO_2_ nanotubes on bone marrow mesenchymal stem cells (BMSCs). They demonstrated that surface nanotopography of TiO_2_ affected regulatory transcription factors such as SREBP1, which influenced osteo- vs. adipogenic differentiation balance. TiO_2_ nanotubes enhanced the expression of osteogenic regulatory factors while suppressing adipogenic genes, thereby supporting MSC osteogenic commitment. This finding highlights another mechanism through which TiO_2_ nanostructures contribute to bone tissue engineering [59]. Liao et al. investigated the effects of Iron oxide NPs (IONPs) labeling on precartilaginous stem cells (PCSCs). They treated PCSCs with iron oxide NPs and found that IONPs were biocompatible and significantly enhanced osteogenic markers such as alkaline phosphatase activity, mineralized nodule formation, and osteogenic gene expression in vitro. Furthermore, when IONPs-labeled PCSCs were incorporated into a printable hydrogel scaffold and implanted in rabbit femoral defects, bone regeneration was significantly accelerated compared to controls, demonstrating that IONPs can improve stem cell-based bone healing [60]. Wu et al. developed hydrogels cross-linked with vinyl-coated iron oxide NPs and tested them in a rat cranial defect model. They found that the inclusion of IONPs strengthened the hydrogel’s mechanical properties and significantly promoted both angiogenesis and osteogenesis compared to hydrogels without NPs. The enhanced vascular structure formation and new bone regrowth in vivo demonstrate the dual regenerative potential of IONP-based composites for complex bone defect repair [61]. Bin et al. incorporated superparamagnetic Fe_3_O_4_ NPs into a poly-L-lactic acid (PLLA) scaffold using selective laser sintering. The study showed that the magnetic scaffold generated micro-magnetic fields that continuously stimulated osteoblast-like MG63 cells, enhancing cell attachment, proliferation, and alkaline phosphatase activity. The results suggest that iron oxide NPs can not only reinforce scaffold mechanics but also provide magnetic stimulation to support bone cell function, a useful property in bone tissue engineering [62]. Lian et al. developed a phosphate-functionalized chitosan hydrogel doped with zinc oxide NPs (CSMP-ZnO) to address critical-sized bone defects. They reasoned that the sustained release of Zn^2+^ ions from ZnO could not only promote osteoblast activity but also stimulate angiogenesis and extracellular matrix formation. In in vitro assays, the ZnO-doped hydrogel significantly increased osteogenic differentiation markers (COL1, RUNX2) and enhanced mineralization compared with the undoped hydrogel. In a rat calvarial defect model, the CSMP-ZnO hydrogel group exhibited greater new bone volume and trabecular number, along with enhanced vascular formation, indicating that ZnO-NPs can improve both osteogenesis and angiogenic support needed for effective bone healing [63]. Zalama et al. investigated the regenerative effect of zinc oxide NPs (ZnONPs) in combination with platelet-rich fibrin (PRF) on large segmental ulnar defects in a rabbit model. They hypothesized that ZnONPs would accelerate bone regeneration beyond the effects of PRF alone. Their findings showed that the PRF + ZnONPs group had a markedly faster reduction in defect size, higher bone density, and improved callus remodeling compared with PRF alone or empty controls, demonstrating that ZnONPs can enhance bone growth and remodeling in critical bone defects [64]. Zhou et al. prepared sodium alginate/hydroxyapatite scaffolds reinforced with ZnO NPs aiming to improve scaffold performance for bone tissue engineering. They found that increasing ZnO content enhanced apatite deposition and bioactivity of the scaffolds, and cell culture studies showed improved osteoblast adhesion and growth compared with scaffolds without ZnO. The improved mechanical properties and in vitro bioactivity suggested that ZnO incorporation can strengthen scaffold osteogenic capacity, making ZnO-NPs promising for bone regeneration applications [65]. Wang et al. fabricated gelatin methacryloyl (GelMA) hydrogels containing CeO_2_ NPs and bone marrow stem cells (BMSCs) to evaluate their effect on bone regeneration. They hypothesized that the antioxidant and immunomodulatory properties of CeO_2_ could improve the healing microenvironment. In vitro, the GelMA-CeO_2_-BMSC hydrogels exhibited good biocompatibility, increased osteogenic differentiation markers, and reduced pro-inflammatory responses by inhibiting M1 macrophage polarization while promoting M2 polarization. When implanted into critical-sized calvarial defects in animal models, the composite hydrogels led to significantly enhanced bone regeneration compared with controls [66]. Purohit et al. developed a gelatin-alginate scaffold incorporated with CeO_2_ NPs to address oxidative stress and enhance bone regeneration. They found that adding nanoceria improved mechanical properties and supported better cell attachment and proliferation. Osteogenic differentiation of mesenchymal stem cells was increased in scaffolds with CeO_2_, as demonstrated by elevated alkaline phosphatase activity and upregulation of osteogenic genes such as RunX2 and osteocalcin. The antioxidant capacity of CeO_2_ also helped scavenge free radicals, creating a more conducive environment for bone tissue formation [67]. Wu et al. investigated the inclusion of CeO_2_ NPs in micro-arc oxidation coatings on biodegradable magnesium alloys for orthopedic use. They found that CeO_2_ NP incorporation stabilized the coating, enhanced biocompatibility, and regulated the degradation profile, reducing inflammation during bone healing. Both in vitro osteogenesis assays and in vivo implantation showed that the CeO_2_-enriched coatings facilitated more robust bone reconstruction and stronger integration between new bone and native tissue, suggesting real potential for CeO_2_ in implantable bone biomaterials [68]. Luo et al. demonstrated that CeO_2_ NPs promote osteogenic differentiation of bone-forming precursor cells by activating intracellular pathways such as ERK signaling. They reported increased mineralization and elevated expression of osteogenic markers, including Runx2 and osteocalcin, in cultures treated with CeO_2_ NPs. In addition, CeO_2_ was shown to enhance angiogenesis by stabilizing HIF-1α and increasing VEGF expression, a key factor in coupling vascularization with bone regeneration [69].

#### 3.1.2. Organic NPs

Organic NPs are primarily derived from natural or synthetic polymers and are widely employed for their flexibility in design, biodegradability, and capacity for controlled delivery of bioactive molecules.

##### Natural Polymer-Based NPs

Natural polymer-based NPs, including those composed of chitosan, gelatin, collagen, and alginate, are inherently biocompatible and often possess intrinsic bioactivity. Their chemical similarity to components of the extracellular matrix allows favorable interactions with both immune cells and osteogenic cells. Chitosan-based NPs, for example, exhibit mild immunostimulatory properties and can influence macrophage activation while supporting osteoblast adhesion. Gelatin and collagen NPs provide cell-adhesive motifs that enhance osteogenic differentiation and matrix deposition. Im et al. engineered 3D chitosan hydrogels reinforced with biomimetic nanocrystalline hydroxyapatite (nHA) and quantified early osteoblast attachment using a short-term adhesion assay. In their 4-h adhesion study, increasing nHA content produced a clear upward trend in adherent osteoblast density. Notably, 20 wt% nHA in chitosan yielded the highest cell attachment, and adhesion on the 20% nHA scaffolds was reported to be ~59% higher than pure chitosan controls, indicating that nanoscale HA incorporation into a chitosan matrix can markedly improve the initial cell–material anchoring phase [70]. Kazimierczak et al. developed highly macroporous chitosan/agarose/nano-hydroxyapatite (nanoHA) cryogel scaffolds and assessed osteoblast adhesion and spreading (MC3T3-E1 and hFOB 1.19). They first demonstrated that these extremely hydrophilic nanocomposites exhibited a strong tendency to adsorb adhesive proteins—especially fibronectin—which is mechanistically consistent with enhanced integrin-mediated attachment. In cell assays, cytoskeletal staining showed osteoblasts on the scaffolds were well spread and formed clear actin–scaffold junctions, supporting the interpretation that nanoHA-reinforced chitosan matrices provide an adhesion-permissive interface through protein-mediated bioactivation of the surface [71]. Soriente et al. developed chitosan/hydroxyapatite nanocomposite scaffolds via sol–gel and freeze-drying and evaluated their effect on cell behavior. They observed that by increasing the nano-HA content, the scaffolds supported enhanced cell adhesion, viability, and osteogenic differentiation toward the osteoblast lineage in human mesenchymal stem cells (hMSCs). The authors noted that higher inorganic filler content correlated with better cell attachment and osteoinductive response, indicating that the nano-HA component plays a critical role in promoting an adhesive and biologically active microenvironment for osteoblast commitment [72]. Verma et al. investigated osteoblast adhesion and proliferation on nanocomposite films composed of chitosan, polygalacturonic acid (PgA), and hydroxyapatite. They found that the nanocomposite formulations significantly enhanced osteoblast adhesion and spreading compared to controls, supporting the view that chitosan-based nanostructured matrices with inorganic additives yield improved biological performance. While not exclusively chitosan NPs, this study highlights the generality of nano-modified chitosan composites in promoting osteoblast attachment [73]. Shi et al. studied chitosan-coated iron oxide NPs and reported that the chitosan coating improved osteoblast viability and differentiation markers in vitro. Transmission electron microscopy showed that chitosan-coated NPs tended to adhere to osteoblast membranes more prominently than uncoated particles, and osteoblast proliferation and phenotype markers were enhanced in the presence of the coated NPs. This suggests that surface modification with chitosan enhances osteoblast membrane interactions and adhesion behavior [74]. Although more recent and focusing on chitosan/HA hydrogels, Di Stefano et al. showed that adding hydroxyapatite NPs to a chitosan hydrogel produced a biocompatible matrix where cell viability and early signs of osteoblast differentiation were observed. While the primary readout was differentiation, the improved cell–matrix interactions and morphological behavior in the presence of NPs imply a supportive influence on cell adhesion as part of the overall cellular response [75]. Mu et al. developed an injectable platelet-rich fibrin (iPRF) hydrogel reinforced with gelatin NPs, forming a double-network (DN) structure in which the fibrin network provides integrity while the GNP colloidal network dissipates mechanical energy. In vitro and in a rabbit sinus augmentation model, the GNP–iPRF DN hydrogel showed enhanced bone-forming performance, consistent with the concept that gelatin’s cell-adhesive motifs (arginine-glycine-aspartic acid (RGD)-like sequences) and ECM-like biochemistry support adhesion-driven osteogenic signaling and subsequent matrix production/mineralization [76]. Yang et al. fabricated a porous titanium scaffold functionalized with zoledronic-acid-loaded gelatin NPs and evaluated both osteogenic activity and the balance of bone formation vs. resorption. Their integrated GNP system was reported to promote a bone-favorable response in vitro and in vivo, consistent with gelatin NPs acting as a biointeractive interface (protein-adsorbing, integrin-engaging surface) that can improve osteoblast-lineage function and support downstream matrix deposition/mineralization alongside the drug effect [77]. Abdulahy et al. reported a gelatin-based nanocomposite scaffold designed for bone regeneration and examined osteogenic outcomes after incorporation of zoledronic acid. Their study showed stronger osteogenic differentiation readouts (typical endpoints include osteogenic gene/protein markers and mineral deposition assays), supporting the interpretation that gelatin-based nanoengineered constructs can provide cell-adhesive biochemical cues (via gelatin’s collagen-derived motifs) and a permissive microenvironment for ECM formation and mineral accumulation [78]. Li et al. engineered an enzyme-cross-linked gelatin NP (GNP)/silk fibroin aerogel incorporating strontium ranelate and evaluated osteogenesis in an OVX-induced osteoporosis setting. The aerogel platform couples (i) gelatin NP-derived ECM mimicry and cell-adhesive sequence availability with (ii) osteoactive strontium signaling, and the authors reported osteogenesis-promoting outcomes, aligning with a mechanism where improved cell anchorage and mechanobiological engagement support osteogenic maturation and matrix production [79]. Dasgupta et al. prepared hydroxyapatite–collagen NPs (reported in the ~50–70 nm size range) and incorporated them (~10 wt%) into a photocrosslinkable polyanhydride paste intended for bone substitution. They then cultured human umbilical cord-derived MSCs on the NP-containing constructs to evaluate cell–material interactions. The authors reported that adding the collagen-bearing nano-phase improved the construct’s mechanical performance and supported cell proliferation and osteogenic differentiation readouts (standard osteogenic markers and mineralization-type endpoints were used to judge osteoinductive behavior in vitro) [80]. Ebrahimi et al. compared 3D-printed Polycaprolactone (PCL) scaffolds with surface modifications including collagen type I and collagen + hydroxyapatite. They reported that collagen-containing coatings improved cell–scaffold interactions and that the collagen/HA condition produced stronger osteogenic differentiation outcomes (typical endpoints include osteogenic marker expression and calcium-mineral readouts). Mechanistically, the collagen layer is often interpreted as providing integrin-mediated adhesion cues that support osteoblastic commitment and matrix production on otherwise bioinert PCL [81]. Calabrese et al. evaluated collagen–hydroxyapatite (including Mg-doped HA) scaffolds with human adipose-derived MSCs. They reported robust osteogenic potential in vitro, supported by Alizarin Red S staining and osteogenic gene-expression profiling, consistent with a material environment where collagen-mediated adhesion and a mineral phase jointly favor osteogenesis and matrix/mineral deposition [82]. Tamaño-Machiavello et al. synthesized acrylate-based substrates functionalized with gelatin (derived from collagen) to present ECM-like adhesive motifs on the surface. They cultured human bone marrow MSCs on these gelatin-grafted biomolecule substrates and reported that the grafting significantly altered gene expression profiles indicative of early osteogenic commitment compared to non-functionalized substrates, consistent with the concept that collagen-derived adhesion cues facilitate osteogenic differentiation signaling in vitro [83]. Oh et al. examined collagen–hydroxyapatite (Col–HA) hydrogel scaffolds and assessed their effect on early bone cell behavior. When osteoprogenitor cells were cultured on these hydrogels, they observed enhanced cell viability, osteogenic differentiation, and early markers of matrix deposition compared with collagen-only controls, suggesting that the fibrillar collagen matrix combined with nanostructured mineral boosts cell adhesion and downstream osteogenic pathways [84]. However, natural polymers may suffer from batch-to-batch variability and limited mechanical stability, which can restrict their standalone use in load-bearing applications.

##### Synthetic Polymeric NPs

Synthetic polymeric NPs, such as those based on poly(lactic-co-glycolic acid), polycaprolactone, and PEG derivatives, offer greater control over physicochemical properties and degradation kinetics. These systems are particularly effective as carriers for osteoinductive factors, immunomodulatory drugs, and nucleic acids. In the context of bone immune modulation, polymeric NPs enable spatiotemporally controlled release of anti-inflammatory agents, cytokines, or gene regulators, thereby preventing prolonged inflammation while supporting subsequent osteogenesis. Importantly, organic NPs can be engineered to interact selectively with immune cells. By tailoring particle size, surface charge, and degradation rate, polymeric NPs can preferentially target macrophages or dendritic cells within the bone defect, influencing cytokine secretion profiles and immune cell recruitment. Such strategies highlight the role of organic NPs not merely as passive carriers but as active regulators of the osteoimmune microenvironment. Zhao et al. incorporated BMP-2-encapsulated PLGA microspheres into a 3D-printed PLGA copolymer/CaSO_4_ scaffold and studied its effect on bone regeneration in vitro and in vivo. Their results showed that the BMP-2/PLGA microspheres enhanced osteogenic differentiation markers (e.g., OSX, ALP) and increased new bone formation in defect models compared to scaffolds without PLGA microspheres. Importantly, the controlled release of BMP-2 from the PLGA carriers provided a sustained osteoinductive signal that boosted osteoblast lineage activity, indicating that PLGA nanoparticulate systems can act as osteoinductive factor reservoirs that support prolonged regenerative stimulation [85]. Long et al. designed 3D-printed composite scaffolds composed of PLGA and black phosphorus (BP) nanosheets and investigated their effects on immune response and bone regeneration in vitro and in a steroid-associated osteonecrosis (SAON) rat model. They found that the PLGA/BP scaffolds significantly recruited macrophages and induced polarization toward the anti-inflammatory M2 phenotype, leading to downregulation of pro-inflammatory markers and promotion of a pro-regenerative microenvironment. In parallel, human bone marrow mesenchymal stem cells (hBMSCs) cultured on these scaffolds exhibited increased proliferation, osteogenic differentiation, and mineralization, with transcriptomic data indicating activation of PI3K-AKT signaling associated with osteogenesis. These results support that PLGA composite nanoscaffolds can actively regulate the osteoimmune niche and thereby enhance bone regeneration outcomes relative to PLGA alone [13]. Gao et al. developed PLGA NPs loaded with 1,25-dihydroxyvitamin D3 (active vitamin D) and incorporated them into a chitosan hydrogel for bone defect application. Although full journal publication details are pending, the research demonstrates that local sustained delivery of 1,25-D3 from PLGA NPs improved bone healing outcomes by modulating inflammatory gene expression and promoting osteoblast differentiation and mineralization in preclinical models. The authors interpreted these results in the context of immune–osteogenic cross-talk, where the anti-inflammatory and pro-osteogenic effects of 1,25-D3 are enhanced by controlled release [86]. Liu et al. fabricated hierarchical composite scaffolds combining PLGA with mesoporous bioactive glass (MBG) and evaluated their effects on macrophage phenotype and bone regeneration. They showed that certain MBG content levels within the PLGA matrix could modulate macrophage polarization toward an anti-inflammatory phenotype, which was accompanied by enhanced angiogenesis and osteogenesis in vivo. This outcome implies a synergy where the PLGA carrier framework regulates immune cell behavior via released ions and structural cues, ultimately improving bone formation over 3D overhaul biomaterials [87]. Song et al. fabricated 3D-printed hydroxyapatite (HA)/PCL composite scaffolds with varied fiber orientations and evaluated their impact on macrophage polarization and osteogenic differentiation of bone marrow mesenchymal stem cells (BMSCs). The results showed that certain fiber arrangements induced macrophage polarization toward the anti-inflammatory M2 phenotype and upregulated osteogenic markers in BMSCs. This immune modulation correlated with enhanced expression of osteogenic genes and greater mineral deposition in vitro, highlighting that PCL scaffolds functionalized with bioactive phases can create an osteoimmune-friendly microenvironment that supports subsequent bone formation [88]. Huang et al. prepared micropatterned 3D-printed PCL scaffolds with grating features and investigated their effect on osteogenic differentiation and macrophage polarization. They found that micro-topographical cues on PCL surfaces significantly increased the osteogenic differentiation of BMSCs while inducing macrophages (RAW264.7) to polarize toward the M2 anti-inflammatory phenotype; pro-inflammatory M1 markers were suppressed accordingly. These outcomes demonstrate that engineering the physical architecture of a PCL biomaterial can modulate immune cell behavior and thereby enhance osteogenesis [89]. Yarahmadi et al. synthesized green-produced copper oxide NPs (CuO NPs) and applied them onto 3D-printed PCL scaffolds to evaluate effects on bone mesenchymal stromal cells. In vitro tests indicated enhanced alkaline phosphatase (ALP) activity and osteogenic differentiation, while in vivo implantation in rat femoral defects showed increased immature bone formation and significant collagen deposition. Although not pure NP delivery, the study demonstrates that PCL scaffolds combined with osteoactive NPs modulate cell behavior and support bone formation [90]. Zhang et al. investigated the effects of PEG-coated gold NPs (GNPs) of different sizes (4, 18, 45 nm) on osteogenic differentiation in pre-osteoblastic MC3T3-E1 cells, human MSCs, and rat MSCs. They observed that NPs of ~18–45 nm exhibited enhanced alkaline phosphatase (ALP) activity, increased mineralized nodule formation, and upregulated osteogenic genes (e.g., Runx2, COL1) compared with smaller particles. Mechanistic analysis implicated activation of the Wnt/β-catenin pathway in the pro-osteogenic effect. In a rabbit femur defect model, 45-nm PEGylated GNPs grafted in hydrogel also promoted new bone formation versus the control hydrogel. This work shows that PEGylation coupled with NP size tuning can directly influence osteogenic differentiation and bone regeneration [91]. Calabrese et al. developed core–shell silica NPs functionalized with PEG, designed to interact optimally with bone cells. In in vitro studies with human adipose-derived MSCs (hADSCs), these PEG-silica NPs enhanced alkaline phosphatase activity, calcium deposition, and matrix mineralization compared with non-functionalized silica controls. The study indicated that modifying NPs with PEG improved cellular interactions and osteoinductive potential, illustrating how physical nanostructure and surface chemistry can support osteogenic differentiation [92].

#### 3.1.3. Hybrid NPs

Hybrid NPs integrate organic and inorganic components within a single system, aiming to combine the structural and bioactive advantages of inorganic materials with the delivery and immunomodulatory capabilities of organic carriers. These systems are increasingly recognized as advanced platforms for bone regeneration, particularly in complex or immunocompromised healing environments.

##### Polymer–Ceramic Hybrid NPs

Polymer–ceramic hybrid NPs are among the most common hybrid designs. In these systems, inorganic cores such as hydroxyapatite or bioactive glass are coated or embedded within biodegradable polymer matrices. This configuration enhances particle stability and dispersion while preserving osteoconductivity and enabling controlled release of therapeutic agents. From an immunological perspective, polymer coatings can mitigate the potential inflammatory effects of inorganic surfaces while allowing fine-tuning of immune cell interactions. Fu et al. engineered sericin/nano-hydroxyapatite (nHA) composite hydrogels (with a graphene-based component) to couple osteoconductivity with immune regulation. They reported that the hybrid formulation shifted macrophage polarization toward a pro-regenerative (M2-like) phenotype, which in turn supported osteogenic activity of mesenchymal stromal cells and improved bone-forming outcomes in defect models [93]. Chen et al. investigated selenium-doped mesoporous bioactive glass (MBG) NPs as immunoregulatory inorganic nano-cues (often integrated into polymeric carriers/scaffolds in translational designs). They showed that Se incorporation could reduce oxidative stress and reprogram macrophage metabolism, promoting a polarization state associated with tissue repair, alongside enhanced bone regeneration in vivo [51]. Chen et al. reported MBG surface-modified PLGA systems designed to improve bioactivity without losing polymer-handling advantages. Their data indicated that MBG functionalization increased osteogenic responses compared with unmodified PLGA constructs, consistent with a design rule that mesoporous glass at the interface can boost cell-instructive cues while PLGA governs structural stability and release behavior [94]. Oudadesse et al. developed chitosan scaffolds incorporating bioactive glass NPs (BGN) using freeze-gelation. They demonstrated that the hybrid promoted bone-regenerative performance, consistent with chitosan’s role in modulating inflammatory reactivity, while glass NPs supply dissolution ions and apatite-forming capacity that drive osteogenesis [95]. Yang et al. proposed a strategy combining nano-hydroxyapatite surfaces with sequentially presented immunomodulatory cytokines to regulate macrophage polarization and improve bone regeneration. While implemented on an implant surface rather than a free NP carrier, the work is frequently cited as mechanistic support for hybrid systems where polymer layers or biomolecule-binding domains are used to time-control immune cues on HA-containing interfaces [96]. Tao et al. presented a PLGA/HA composite design for bone defect repair and reported improved regenerative outcomes in vivo, supporting the translational rationale for pairing PLGA (handling, degradability) with HA (osteoconductivity). Although scaffold-form, the findings align with the same interfacial principles used in PLGA-coated HA nanoparticulate systems [97].

##### Lipid–Polymer and Polymer–Metal Hybrid Systems

Lipid–polymer and polymer–metal hybrid NPs have also been explored for multifunctional bone therapies. These systems enable simultaneous delivery of osteogenic and immunomodulatory signals, such as growth factors combined with anti-inflammatory drugs or gene regulators. By addressing multiple biological pathways in parallel, hybrid NPs are particularly well suited to modulate the dynamic and stage-dependent nature of bone regeneration. Recent bone-targeted NP systems further demonstrate that surface decoration with mineral-affinitive ligands, such as alendronate or aspartic acid-based peptides, can improve localization of therapeutic nanocarriers within bone tissue [20,98]. In addition, hybrid NPs facilitate the design of stimuli-responsive systems that adapt to changes in the bone microenvironment, such as pH shifts or enzymatic activity associated with inflammation. This responsiveness allows therapeutic payloads to be released selectively during specific phases of healing, thereby aligning immune modulation with osteogenic progression. Briffault et al. developed lipid–polymer hybrid NPs (LPNPs) loaded with an SFRP1-silencing GapmeR and compared dose and targeting strategies to treat impaired bone formation in osteoporosis. They reported that dual functionalization (e.g., combining bone-targeting moieties) improved in vivo performance, and that therapeutic efficacy was strongly dose- and targeting-dependent, supporting LPNPs as a practical format for stage-aware gene regulation in compromised bone healing [21]. Liu et al. designed a bone resorption-surface-targeting liposome system by conjugating an Asp8 peptide to preferentially bind mineral surfaces enriched at osteoclast-active sites. By encapsulating an antagomir (antagomir-148a) to suppress osteoclastogenic signaling, they demonstrated that lipid nanocarriers can be engineered to localize nucleic-acid therapeutics to specific remodeling microdomains, enabling immuno-skeletal modulation through osteoclast pathway control [99]. Liu et al. reported bone-targeted lipid NPs delivering Runx2 mRNA bearing m7G methylation, aimed at boosting osteoblast lineage commitment under senile osteoporosis. Their results indicated that targeted lipid nanocarriers can enable potent anabolic (osteogenic) signaling at the gene-expression level, highlighting how lipid-based systems can be paired with molecular engineering of cargo to strengthen bone formation in age-compromised settings [100]. Gan et al. presented a prototypical microenvironment-responsive co-delivery NP concept relevant to “stage-dependent” healing: chitosan-functionalized mesoporous NPs that release BMP-2 rapidly (outer polymer domain) and then provide pH-triggered intracellular release of dexamethasone (mesopores). They showed that sequential presentation can align an early osteoinductive cue with a later anti-inflammatory / pro-osteogenic pharmacologic signal, illustrating how hybrid architectures can synchronize immune modulation with osteogenesis via pH-responsiveness [101]. Fu et al. fabricated a mussel-inspired polymer/metal hybrid construct incorporating gold NPs within a polymeric matrix (with adhesive/catechol chemistry), and reported significantly enhanced osteoblast behaviors (adhesion, proliferation, osteogenic differentiation) alongside improved mineralization outcomes. Although implemented as a composite construct, the work is widely used to justify polymer–Au nano-additives as multifunctional components that can couple cell-instructive surface chemistry with mechanical/structural support [102]. Wu et al. developed a polymer network cross-linked by iron oxide NPs, creating a hybrid material where the metal oxide nanophase contributes bioactivity and the polymer phase provides formability and retention. In a rat cranial defect model, they observed improved vascular regeneration and bone regrowth, consistent with the broader strategy of using iron-oxide-based hybrids to integrate regenerative signaling with platform-level functions (e.g., imaging or magnetic responsiveness in related designs) [61]. Tan et al. reported a 3D-printed PLGA-based composite incorporating MgO-related components and a bioinspired surface chemistry layer, emphasizing macrophage-linked mechanisms. Their results supported a model in which magnesium-associated cues contribute to macrophage polarization pathways that subsequently enhance stromal osteogenesis, aligning polymer–metal oxide composites with immune microenvironment engineering rather than purely structural graft roles [103]. Sullivan et al. demonstrated matrix-metalloproteinase (MMP)-responsive micellar NPs for enzyme-triggered drug liberation (a design principle highly transferable to inflamed bone defects where proteases are elevated). Their data showed that enzymatic responsiveness can be used to gate exposure of therapeutics preferentially within diseased/inflamed tissues, supporting the general strategy of protease-cued release for spatiotemporal control [104].

Overall, the classification of NPs into organic, inorganic, and hybrid categories provides a useful framework for understanding their distinct and complementary roles in bone regeneration. As the field moves toward more sophisticated osteoimmunomodulatory strategies, hybrid systems that integrate structural support, controlled delivery, and immune regulation are likely to play an increasingly central role in next-generation bone regenerative therapies (Table 2).

To further highlight recent advances in osteoimmunomodulatory nanomedicine, Table 3 provides a comparative summary of representative research studies published between 2020 and 2026. The selected studies encompass a broad spectrum of NP platforms, including metallic, ceramic, polymeric, lipid-based, and hybrid systems, and compare their physicochemical characteristics, immune-regulatory mechanisms, cellular targets, and regenerative outcomes. This comparison illustrates how rational NP design can influence specific immune pathways while simultaneously promoting osteogenesis, angiogenesis, and functional bone regeneration.

**Table 2:** Surface Engineering Strategies for Immune Regulation in Bone Regeneration.

Surface Engineering Strategy	Substrate	Immune Regulation Outcome	Bone Regeneration Outcome	Reference
Layer-by-layer immobilization of IL-4 and RGD peptides on Polydopamine (PDA)-modified TiO_2_ nanotubes	Titanium implant	Promoted M2 macrophage polarization (↑ IL-10), reduced pro-inflammatory signaling	Enhanced MSC osteogenic differentiation via BMP/Smad/Runx2 activation	[105]
IL-4-assisted Ca–Sr–Zn–phosphate conversion coating	Titanium implant	Controlled M2 macrophage polarization; reduced inflammatory cytokines	Improved osseointegration and new bone formation	[106]
Sr–Zn phosphate chemical conversion coating with optimized ion release	Titanium implant	Induced M2 macrophage phenotype; enhanced pro-osteogenic cytokine expression	Accelerated femoral defect healing and bone–implant integration	[107]
Surface potential modulation via polydopamine coating	Titanium surface	Shifted macrophages toward M2 phenotype through FAK–PI3K–Akt–mTOR signaling	Enhanced in vivo osseointegration	[108]
pH-responsive hydrogel coating enabling sequential Sophoridine (SOP) → Luteolin (LUT) release	Titanium implant	Sequential M1-to-M2 macrophage transition (PI3K–AKT pathway involvement)	Improved rat femoral defect osseointegration	[109]
Antimicrobial peptide (GL13K) surface functionalization	Titanium surface	Modulated macrophage polarization and reduced inflammatory markers	Supported osteogenic microenvironment and implant integration	[110]
Osteoimmunomodulatory “biopatch” surface interface engineering	Bone defect interface	Regulated osteoimmune microenvironment; reduced inflammatory activation	Promoted in vivo bone regeneration	[111] (Figure 4D)
ROS-responsive titanium implant coating	Titanium implant	Promoted M2 polarization; suppressed osteoclastogenesis	Enhanced bone integration and regeneration	[112]
Hydrogel coating incorporating selenium NPs (SeNPs) and lithium	Titanium substrate	Scavenged excess ROS; regulated inflammatory signaling	Supported pro-regenerative immune environment and bone regeneration	[113]
Biointerface membrane coating for osteoimmune niche formation	Bone graft/defect interface	Established pro- regenerative osteoimmune microenvironment	Enhanced in situ osteogenesis and new bone formation	[114] (Figure 3)

**Table 3:** Comparative overview of research studies on immunomodulatory NPs for bone regeneration published between 2020 and 2026.

NP/System	Physicochemical Characteristics	Immunomodulatory Mechanism	Target Immune Cells	Regenerative Outcome	Reference
Biomimetic anti-inflammatory nanocapsule	Biomimetic polymeric nanocapsule; cytokine-blocking design	Blocks pro-inflammatory cytokines and induces M2 polarization	Macrophages	Enhanced bone tissue repair	[115]
Nanosilver–halloysite nanotube/GelMA hydrogel	Ag-loaded halloysite nanotubes in GelMA	Antibacterial action with osteoimmunomodulatory activity	Macrophages	Improved antibacterial bone regeneration	[116]
Graphene-modified CePO_4_ nanorods	ROS-regulating cerium phosphate nanorods	Reduces oxidative stress and regulates macrophage phenotype	Macrophages	Improved osteoinductive activity	[117]
Gold NPs	Size-dependent AuNPs	Induces M2 polarization and anti-inflammatory cytokine profile	Macrophages	Restored bone formation in mice	[118]
Dual-targeted baicalein nanoplatform	Bone/macrophage-targeted nanocarrier	Delivers baicalein to promote M2 polarization	Macrophages	Enhanced fracture healing	[119]
Immunomodulatory layered double hydroxide NPs	LDH nanosheets/NPs	Regulates inflammatory signaling and immune microenvironment	Macrophages	Improved osteogenesis and bone regeneration	[120]
Chitosan/silk fibroin/cellulose NP scaffold	3D-printable natural-polymer nanocomposite	Drives M2 macrophage polarization	Macrophages	Enhanced bone regeneration	[121]
Amphiphilic nanomedicine-loaded fibrous scaffold	Drug-loaded amphiphilic nanomedicine in fibrous scaffold	Spatiotemporal osteoimmunomodulation	Macrophages	Improved osteogenesis and vascularized repair	[122]
Tantalum NPs	Inorganic metallic NPs	Modulates macrophage inflammatory response	Macrophages	Supports osteogenic microenvironment	[123]
Phosphatidylserine liposome multilayers	Bioinspired liposomal multilayer coating	Mediates M1-to-M2 macrophage transition	Macrophages	Enhanced bone tissue regeneration	[124]
Conductive GO/nHAp scaffold	Polydopamine-mediated graphene oxide and nano-hydroxyapatite	ROS scavenging; suppresses M1 and activates M2 phenotype	Macrophages	Accelerated diabetic periodontal bone regeneration	[125]
Lipid NP-assisted miR-29a delivery	Core–shell lipid nanocarrier	Regulates collagen synthesis and NF-κB activation	Macrophages/osteogenic cells	Promoted osteogenesis and bone regeneration	[126]
Silica nanocarrier with rapamycin	Mesoporous silica nanocarrier	Promotes autophagy-mediated M2 polarization	Macrophages	Regulated bone regeneration	[127]
PLGA/black phosphorus scaffold	3D-printed PLGA scaffold with BP nanosheets	Regulates osteoimmune microenvironment; promotes M2 phenotype	Macrophages	Enhanced osteogenesis and bone regeneration	[13]
Anti-inflammatory/osteoinductive NP hydrogel	Composite hydrogel carrying immunomodulatory and osteogenic NPs	Reduces inflammation while supporting osteogenic signaling	Macrophages	Effective bone regeneration	[128]
Size-dependent gold NPs	AuNPs with controlled particle size	Size-dependent macrophage polarization	Macrophages	Enhanced osteoimmunomodulatory bone regeneration	[129]
Bone-targeted NP drug delivery system	Bone-targeting nanocarrier	Local osteoimmunomodulation at fracture site	Macrophages	Improved fracture healing	[130]
Se-nHA/PC composite microsphere	Selenium-doped nano-hydroxyapatite/polymer microsphere	Regulates macrophage polarization and inflammation	Macrophages	Promoted periodontal bone regeneration	[131]
GO/Cu nanosheet-integrated hydrogel	Graphene oxide/copper nanosheet hydrogel	Anti-inflammatory and pro-angiogenic immune regulation	Macrophages	Enhanced calvarial bone regeneration	[132]
SPION/PLGA scaffold	Infection-sensitive magnetic NP/polymer scaffold	Infection-responsive immune regulation	Macrophages	Promoted infected bone regeneration	[133]
Ultra-low-dose AgNP-loaded TiO_2_ nanotubes	Silver NP-loaded nanotubular titanium surface	Modulates osteoclast activity and immune response	Macrophages/osteoclast-lineage cells	Improved osteoporotic bone regeneration	[134]
ROS-triggered NP hydrogel	ROS-responsive hydrogel co-delivering antibacterial and anti-inflammatory NPs	ROS scavenging and inflammation reduction	Macrophages	Reduced alveolar bone loss in periodontitis	[135]
Intelligent magnetic scaffold	Magnetic scaffold targeting macrophage mitochondria	Mitochondrial immune modulation	Macrophages	Enhanced immune modulation and bone regeneration	[136]
Calcium phosphate NP-immobilized scaffold	CaP NP-functionalized regenerative scaffold	Regulates diabetic inflammatory microenvironment	Macrophages	Improved diabetic bone regeneration	[137]

### 3.2. Surface Chemistry and Functionalization

Beyond material composition, the surface chemistry of NPs plays a decisive role in dictating their interactions with biological systems. Surface properties influence protein adsorption, cellular uptake, immune recognition, and ultimately the regenerative outcome. Rational surface functionalization strategies are therefore essential for optimizing NP-mediated modulation of the osteoimmune microenvironment.

#### 3.2.1. Surface Charge and Hydrophilicity

Surface charge is a key parameter governing NP–cell interactions. Positively charged NPs tend to exhibit enhanced cellular uptake due to electrostatic interactions with negatively charged cell membranes. However, excessive positive charge can also trigger cytotoxicity or undesired inflammatory responses. In contrast, neutral or mildly negative surfaces often demonstrate improved biocompatibility and prolonged circulation time. Hwang et al. reported that when comparing otherwise similar NPs of different surface charges, positively charged particles exhibited significantly higher internalization in HeLa cells than negatively charged ones—a direct experimental confirmation of the role of positive charge in cell uptake efficiency [138]. Öztürk et al. described trends across multiple particle types showing that surface charge strongly influences cellular uptake, with more positively charged particles generally being taken up more efficiently by mammalian cells, although particle size and other features also contribute [139]. Zhang et al. used upconversion NPs to show that a higher surface charge is a primary factor determining increased endocytosis, especially for small NPs, providing single-particle microscopy evidence of charge-driven internalization [140]. Zheng et al. compared negative vs. positive charged quantum dots and showed that negatively charged QDs exhibited lower cytotoxicity, highlighting that surface charge influences toxicity profiles [141].

Hydrophilicity similarly affects biological performance. Hydrophilic surface coatings, such as PEG, can reduce nonspecific protein adsorption and limit recognition by the mononuclear phagocyte system. Abuchowski et al. covalently attached PEG to a model enzyme (bovine liver catalase) and reported that PEG modification reduced immunogenicity while prolonging circulating lifetime compared with the unmodified protein, supporting the concept that a hydrated polymer shell can mask biomolecular surfaces from biological recognition [142]. PEG-coated polymeric nanocarriers can markedly extend blood residence by limiting RES/MPS clearance. Gref et al. introduced biodegradable polymeric nanospheres engineered to be long-circulating and showed that PEG-based surface engineering could yield substantially prolonged circulation, consistent with diminished opsonization-driven phagocytic clearance and reduced sequestration by RES organs [143]. Conformation and packing density of PEG chains critically tune anti-fouling and phagocyte interactions. Li et al. demonstrated that, even for low-protein-affinity nanocarriers, cellular interactions depend strongly on PEG chain density, molecular weight, and conformation. They showed that densely packed PEG in a “brush” regime reduced nonspecific uptake by immune cells (e.g., dendritic cells) compared with more weakly packed “mushroom/collapsed” conformations, linking PEG architecture to reduced phagocytic uptake and improved stealth behavior [144].

In bone regeneration applications, this can help maintain NP stability while enabling controlled interactions with target cells in the defect site.

#### 3.2.2. Protein Corona Formation and Immune Recognition

Upon exposure to physiological fluids, NPs rapidly adsorb proteins, forming a so-called protein corona that defines their biological identity. The composition of this corona is strongly influenced by surface chemistry and can significantly affect immune cell responses. González-García et al. investigated how controlled surface functionalization alters protein corona formation and subsequent macrophage responses. They tailored silica NPs with different chemical groups (amine, carboxylic acid, oxazoline, alkane) and incubated them in human serum/plasma. Their proteomic analysis revealed that corona composition varied significantly with surface chemistry, leading to differential levels of complement proteins and lipoproteins. NPs with carboxylic acid surfaces induced higher pro-inflammatory cytokine production, whereas amine-rich coatings correlated with anti-inflammatory marker expression, demonstrating that surface design can direct physiological outcomes [145]. Lundqvist et al. examined how variations in NP size and surface chemistry influence plasma protein adsorption profiles. Through mass spectrometry-based proteomic analysis, they demonstrated that small differences in surface characteristics significantly altered the relative abundance of adsorbed immunoglobulins and complement proteins. These results provided early experimental evidence that surface chemistry directly modulates immune recognition via selective protein enrichment in the corona [146]. Tenzer et al. performed high-resolution time-resolved proteomic analysis to characterize early corona formation events. They showed that protein adsorption occurs within seconds after plasma exposure and that immune-related proteins are rapidly enriched in the corona. Furthermore, they reported that NP-induced complement activation could occur at very early stages, suggesting that initial corona composition plays a decisive role in acute immune responses [147].

In the bone microenvironment, protein corona formation may modulate macrophage activation, cytokine secretion, and downstream osteogenic signaling. Surface functionalization strategies that promote the adsorption of pro-regenerative proteins, or that minimize binding of pro-inflammatory mediators, can thus steer immune responses toward tissue repair. Understanding and controlling protein corona dynamics is increasingly recognized as a crucial aspect of NP design for immunomodulatory bone therapies. Zhu et al. investigated protein corona formation on hydroxyapatite (HA) versus magnetic hydroxyapatite (MHA) scaffolds under in vitro and in vivo exposure conditions. They reported that the MHA-associated corona was enriched in functional proteins capable of engaging signaling pathways, and mechanistically linked this adsorbed layer to activation of MAPK/ERK signaling in osteoblast-like cells, resulting in enhanced proliferation. This study provided direct evidence that, in a bone-biomaterial context, the acquired protein layer can act upstream of canonical pro-regenerative signaling rather than being a passive coating [148]. Zhu et al. developed an in vivo dynamic protein corona model for magnetic NP (MNP)-infiltrated bone regeneration scaffolds, aiming to connect time-evolving corona composition with inflammatory state and healing outcomes. Their proteomic profiling indicated time-dependent enrichment of proteins associated with immune/inflammatory processes, extracellular matrix regulation, wound healing, and signaling within the corona. Importantly, they concluded that corona dynamics tracked with the inflammatory reaction was predictive of osteogenesis during bone wound healing, supporting the concept that controlling early adsorption events can steer osteoimmune trajectories in vivo [149]. Sun et al. engineered magnetic hydroxyapatite scaffolds and explicitly analyzed macrophage regulation from the perspective of protein corona + intracellular signaling. Using combined proteomics and genomics, they reported that magnetic cues were associated with corona shifts (e.g., differential adsorption of proteins linked to hormone responsiveness vs receptor-linked signaling) and with macrophage pathway changes characterized by upregulated PPAR signaling, suppressed JAK–STAT activity, and enhanced fatty-acid metabolism, collectively favoring M2 polarization. In vivo, they observed macrophage phenotype shifts compatible with a pro-repair immune environment and improved osteogenic outcomes, positioning corona composition as a mechanistic bridge between material cues and osteoimmune regulation [150]. Wang et al. studied how the phase composition of porous calcium phosphate ceramics influences protein adsorption behavior and osteoinduction in vivo (mouse model). Under competitive adsorption conditions, they found strong affinity for BMP-2 across ceramics, and showed that a specific biphasic composition exhibited higher osteoinductive potential after implantation, accompanied by higher local expression of osteogenic markers (e.g., BMP-2 and osteocalcin) in histological analyses. This work supports the idea that tuning surface/phase chemistry can shape a locally pro-osteogenic “adsorption layer” (corona-like interface) that amplifies regenerative signaling in vivo [151]. Mahon et al. developed bone-mimetic nano-hydroxyapatite-containing constructs designed to bias the post-implant immune response toward regeneration. They reported that incorporation of bone-mimetic NPs promoted M2-skewed macrophage responses, increased IL-10-dependent signaling, and enhanced MSC osteogenesis and bone formation. While the paper is framed around osteoimmunomodulation, its implications align closely with corona principles: bone-mimetic surfaces/interfaces in physiological fluids can preferentially recruit or present pro-repair cues that then propagate through macrophage-derived cytokines to osteogenic programs [152]. Zhan et al. reported a nanomedicine strategy that effectively pre-configures the biological interface by using fibronectin-coated polymer NPs (a deliberate, biomimetic “corona-like” surface) combined with payloads targeting oxidative stress and inflammatory cytokines. In a bone-associated inflammatory setting (osteoarthritis), they showed that this multi-modal design could attenuate inflammatory mediators while supporting pro-regenerative tissue responses, illustrating how intentional surface protein presentation can steer local immune signaling in the bone/joint microenvironment [153].

#### 3.2.3. Biofunctionalization with Ligands and Biomolecules

Functionalization with bioactive ligands enables NPs to actively engage specific cellular receptors. Peptides derived from extracellular matrix proteins, such as RGD motifs, can enhance osteoblast adhesion and differentiation. Similarly, functionalization with growth factors or cytokines allows NPs to locally deliver osteoinductive or immunoregulatory signals. In the context of osteoimmunology, NPs functionalized with anti-inflammatory agents or immune checkpoint-modulating molecules can influence macrophage polarization, dendritic cell maturation, and T-cell responses. By promoting a shift from pro-inflammatory to pro-regenerative immune phenotypes, such strategies create a microenvironment conducive to bone regeneration. Zheng et al. fabricated TAT & RGD peptide-modified, naringin-loaded lipid NPs to enhance cell interaction and intracellular delivery in human dental pulp stem cells. They reported that peptide-functionalized NPs improved osteogenic outcomes (e.g., osteogenesis-associated readouts) relative to non-targeted formulations, and supported mechanistic interpretation using metabolomics, consistent with ligand-guided nanosystems amplifying osteogenic differentiation programs [154]. Sitasuwan et al. used RGD-conjugated rod-like viral NPs immobilized on 2D substrates to engage integrin-mediated adhesion of mesenchymal stem cells. They found that RGD-NP presentation enhanced osteogenic differentiation compared with controls, supporting the concept that ECM-mimetic peptide ligands on nanoscale features can upregulate osteolineage commitment through receptor engagement and downstream signaling [155]. Li et al. reported layer-by-layer assembly of IL-4 and RGD on TiO_2_ nanotube surfaces to simultaneously provide an anti-inflammatory cytokine cue and an adhesive integrin-binding motif. They showed that combining IL-4 with RGD created a more favorable early osteoimmune microenvironment (reduced inflammatory bias), and this immune conditioning aligned with improved osteogenic performance, indicating synergistic control of immune–osteogenic coupling by dual biofunctionalization [105]. Li et al. engineered a 3D-printed calcium silicate/β-TCP scaffold loaded with IFN-γ designed to orchestrate a temporal immune sequence (early M1-like activation followed by M2-like reparative polarization driven by material/ionic cues). They reported enhanced angiogenesis-linked outcomes and improved bone-relevant regenerative performance, supporting the osteoimmunology paradigm that appropriately staged immune programming can increase vascularization and osteogenesis in large-defect repair strategies [156]. Mahon et al. showed that bone-mimetic nano-hydroxyapatite particles promoted M2-skewed macrophage polarization and selectively increased IL-10, which they identified as a functional mediator of macrophage-driven enhancement of MSC osteogenesis. In a rat femoral defect model, nano-scale HA functionalization was associated with a more pro-regenerative immune profile (including reduced pro-inflammatory features compared with micro-scale HA) and increased bone volume, demonstrating that nano-biointerfaces can steer innate immunity to support bone regeneration [152]. Jin et al. designed a biomimetic hierarchical intrafibrillarly mineralized collagen nanointerface and evaluated its immunomodulatory and regenerative effects in critical-sized defects. They reported robust bone formation accompanied by increased M2 macrophage polarization and recruitment of host MSCs. Mechanistically, they implicated IL-4-associated signaling in the macrophage–MSC coupling that promoted osteogenic differentiation, and showed that perturbing this axis reduced the pro-osteogenic effect, supporting a causal osteoimmune mechanism [157]. Zhang et al. reported a Ni-MOF-based engineered system aimed at regulating macrophage programs relevant to bone homeostasis and angiogenesis, with an explicitly described link to PD-L1 activation. The work is relevant to “immune checkpoint-modulating” strategies in bone contexts because it frames checkpoint-associated signaling as part of the macrophage regulatory circuitry influencing bone loss prevention and vascular/repair-associated outcomes [158].

#### 3.2.4. Stimuli-Responsive Surface Modifications

Advanced surface engineering approaches have introduced stimuli-responsive functionalities that enable NPs to adapt to the local microenvironment. pH-sensitive, enzyme-responsive, or redox-responsive coatings allow for site-specific release of therapeutic payloads in response to inflammatory or osteogenic cues. For example, NPs designed to release anti-inflammatory agents under acidic conditions can selectively target inflamed bone defects. These smart surface modifications offer a means to synchronize immune modulation with the dynamic phases of bone healing. By responding to endogenous signals, stimuli-responsive NPs can provide precise temporal control over therapeutic interventions, thereby enhancing regeneration while minimizing off-target effects. A growing body of work illustrates the utility of pH-responsive coatings in bone regeneration. For example, Han et al. reported pH-sensitive polylactic acid/chitosan NPs conjugated with osteoprotegerin via hydrazone linkages; these systems demonstrated enhanced release of osteoprotective cargo under acidic conditions representative of inflammatory bone defects, suppressing osteoclastogenesis and promoting osteoblastic differentiation in vitro and in vivo [159]. Similarly, Zeng et al. developed a gelatin NP core carrying bone morphogenetic protein-9 (BMP-9), functionalized with a pH-adaptive metal phenolic network that responded to acidic osteomyelitic microenvironments to release BMP-9 preferentially at the injury site, resulting in improved bone regeneration outcomes [160]. In addition to pH triggers, enzyme-responsive systems have been engineered to capitalize on elevated proteolytic activity during bone remodeling. Lavrador et al. comprehensively describe NPs incorporating enzyme-cleavable linkages that facilitate drug release in response to specific proteases, thus fine-tuning therapeutic availability in dynamic microenvironments such as sites of chronic inflammation or implant integration [161]. More targeted enzyme sensitivity can be achieved by embedding peptide sequences recognized by matrix metalloproteinases (MMPs), a strategy that has been exploited for controlled growth factor delivery in regenerative settings. Redox-responsive nanomaterials are increasingly exploited to achieve microenvironment-triggered therapeutic control in inflammatory bone lesions, where excessive ROS not only sustains M1-like macrophage polarization but also impairs osteogenic differentiation and matrix maturation. To directly couple immune regulation with regeneration, Huang et al. developed a ROS-responsive hydrogel enabling on-demand release of dimethyl fumarate (DMF). In a large bone defect setting, their ROS-labile network provided a dual function—attenuating oxidative/inflammatory burden while supporting osteogenesis—leading to improved bone regeneration alongside suppression of key pro-inflammatory cytokines (e.g., TNF-α, IL-6, IL-1β) relative to controls [162]. Beyond hydrogel depots, redox-triggerable designs are also being integrated into clinically motivated, infection/inflammation-associated craniofacial contexts. Chen et al. reported a ROS-sensitive injectable polysaccharide-based hydrogel incorporating antibacterial TA@Ag NPs, engineered with ROS-sensitive dynamic crosslinking (phenylborate chemistry) and catechol motifs for tissue adhesion. In their study, the platform was designed to simultaneously address infection control, oxidative stress regulation, and macrophage phenotype modulation, ultimately supporting inflammatory mastoid bone regeneration in a challenging otologic microenvironment [163]. Increasingly, investigators are moving from single-trigger constructs to multimodal (dual) responsiveness to better match the temporal sequence of bone healing—early immune dampening followed by pro-regenerative signaling. In a representative example, Ding et al. designed a pH/ROS-responsive magnesium scaffold using hydrazone-bond functionalization to control doxorubicin release for localized postoperative bone tumor therapy while maintaining scaffold function. Notably, the responsive chemistry minimized drug leakage under physiological conditions while enabling substantial release under acidic, tumor-like conditions, illustrating how dual-trigger platforms can sharply improve spatial/temporal control of payload availability in bone-associated pathologies [164]. Finally, multi-stimuli concepts are also being implemented to coordinate inflammation control with preservation of musculoskeletal repair processes. Yang et al. developed an inflammation-responsive hydrogel spray incorporating curcumin-loaded ZIF-8@CeO_2_ NPs, designed to prevent trauma-induced heterotopic ossification via “dual-homeostatic modulation” without compromising normal tissue healing—underscoring how responsive nanocomposites can be tuned to suppress pathological osteogenesis while maintaining regenerative capacity [165]. Collectively, these studies demonstrate that redox- and multi-stimuli-responsive material interfaces can function as adaptive therapeutic actuators in the osteoimmune microenvironment. By coupling endogenous triggers (ROS, acidic pH, inflammatory signals) to precisely timed release and microenvironment conditioning, such systems can reduce early inflammatory damage while enabling later-stage osteogenesis—thereby improving regenerative efficacy and minimizing collateral exposure.

## 4. Mechanisms of Immune Modulation

Bone regeneration is inherently linked to the immune response, as inflammation represents the initial and indispensable phase of the healing cascade. However, the quality, magnitude, and duration of immune activation critically determine whether the regenerative process proceeds toward functional tissue restoration or culminates in fibrotic encapsulation and impaired healing. In this context, the concept of osteoimmunology has emerged to describe the reciprocal interactions between immune cells and skeletal cells within the bone microenvironment. NPs offer unique opportunities to modulate immune mechanisms involved in bone regeneration due to their tunable size, surface chemistry, and capacity for targeted and controlled delivery. Rather than acting solely as passive biomaterials, NPs can actively influence immune cell recruitment, activation, and phenotype. Through direct interactions with immune cells or indirect modulation via released ions and bioactive molecules, NPs orchestrate a dynamic immune landscape that supports osteogenesis. Among immune cell populations, macrophages, T lymphocytes, and dendritic cells play particularly critical roles in shaping the inflammatory and regenerative milieu. The following sections focus on the mechanisms by which NP-based systems regulate macrophage polarization and adaptive immune cell interactions to promote bone regeneration.

### 4.1. Macrophage Polarization

**Figure 3:**
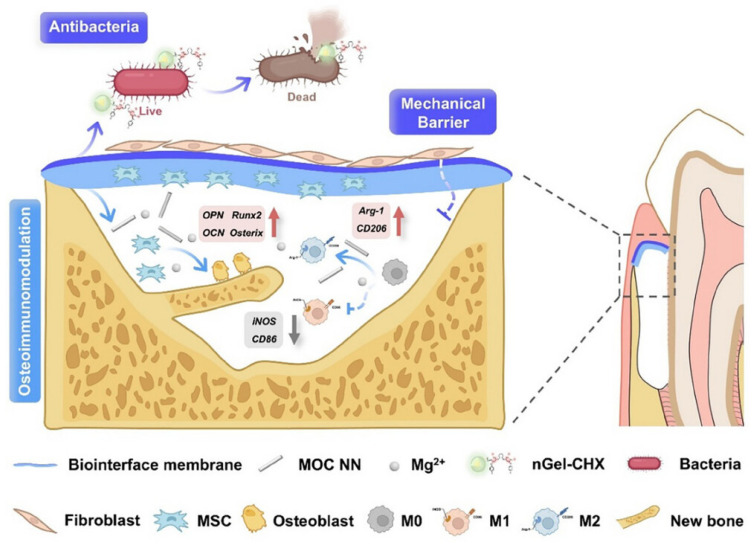
Schematic of the application of the biointerface membrane for treating periodontal defects and the mechanism of membrane-mediated defect repair. MOC NN: nanoneedle-shaped magnesium oxychloride ceramic colloids. nGel-CHX: chlorhexidine-conjugated nanogel. MSC: mesenchymal stem cell. M0: M0 macrophage. M1: M1 macrophage. M2: M2 macrophage [114].

Macrophages are central regulators of bone healing and represent one of the earliest immune cell populations recruited to the site of injury. Upon tissue damage, circulating monocytes infiltrate the bone defect and differentiate into macrophages, which dynamically adopt distinct functional phenotypes in response to local cues. Classically activated macrophages (often referred to as M1-like) are associated with the secretion of pro-inflammatory cytokines and ROS, whereas alternatively activated macrophages (M2-like) support tissue repair, angiogenesis, and extracellular matrix remodeling. A timely transition from an M1-dominated inflammatory phase to an M2-dominated regenerative phase is essential for successful bone regeneration (Figure 3). NPs have demonstrated a remarkable ability to regulate macrophage polarization through multiple mechanisms. Particle size, shape, stiffness, and surface chemistry all influence macrophage uptake and intracellular signaling. Nanoscale materials are preferentially internalized by macrophages via phagocytosis or endocytosis, making these cells particularly responsive to NP-based interventions. Once internalized, NPs can modulate signaling pathways such as NF-κB, MAPK, and STAT, thereby influencing cytokine secretion profiles and macrophage phenotype. Inorganic NPs, particularly calcium phosphate and bioactive glass systems, exert immunomodulatory effects through ion release. Elevated local concentrations of calcium ions have been shown to suppress excessive pro-inflammatory signaling while enhancing the expression of markers associated with reparative macrophages. Similarly, silicon-containing NPs can attenuate inflammatory cytokine production and promote the secretion of vascular endothelial growth factor and transforming growth factor-β, both of which are critical for angiogenesis and osteogenesis. These effects highlight the ability of inorganic NPs to couple immune modulation with downstream regenerative processes. Organic NPs offer additional control over macrophage behavior by enabling the delivery of immunoregulatory molecules. Polymeric NPs encapsulating anti-inflammatory drugs, cytokines, or nucleic acids can selectively alter macrophage polarization at different stages of healing. For example, the sustained release of anti-inflammatory agents can prevent prolonged M1 activation, which is known to inhibit osteoblast differentiation and mineralization. Importantly, organic NPs can be engineered to degrade in response to inflammatory conditions, ensuring that immune modulation occurs in a temporally controlled manner. Surface functionalization further enhances the capacity of NPs to regulate macrophage polarization. NPs modified with specific peptides or polysaccharides can engage macrophage receptors, such as scavenger receptors or toll-like receptors, thereby influencing downstream immune signaling. Subtle variations in surface charge and hydrophilicity can also determine whether NPs elicit pro-inflammatory or pro-regenerative responses. As such, macrophage polarization is not dictated by a single material parameter but emerges from the integrated physicochemical profile of the NP system. Crucially, macrophage-mediated immune modulation directly affects osteogenic cell behavior. Pro-regenerative macrophages secrete factors that enhance mesenchymal stem cell recruitment, proliferation, and differentiation, as well as promote coupling between angiogenesis and osteogenesis. By steering macrophage polarization toward a reparative phenotype, NP-based systems establish an immune microenvironment that actively supports bone formation rather than merely tolerating implanted materials. A number of experimental studies provide concrete evidence that NP composition, size, and interface design can deliberately steer macrophage polarization and thereby improve osteogenic outcomes. Mahon et al. demonstrated that bone-mimetic nano-hydroxyapatite (nanoHA) incorporated into an ECM-based scaffold preferentially drove human macrophages toward an M2-like phenotype, with a marked increase in IL-10 production (linked to c-Maf activation). Importantly, conditioned macrophages enhanced MSC osteogenesis in an IL-10-dependent manner, and in a rat femoral defect model the nanoHA-functionalized scaffolds promoted M2-associated signatures, vascularization, and higher bone volume compared with micron-sized HA controls [152]. Using an “instructive cytokine delivery” strategy, Zhao et al. developed an IL-4-loaded heparin hydrogel that polarized macrophages toward a reparative phenotype and increased TGF-β1/Smad signaling, thereby enhancing BMSC osteogenic differentiation. This work illustrates how biomaterial-based cytokine presentation can be tuned to push macrophage phenotype transitions that are pro-regenerative rather than persistently inflammatory [166]. Beyond cytokine delivery, Patel et al. fabricated 3D-printable chitosan/silk fibroin scaffolds reinforced with cellulose NPs and observed a significant shift from M1 → M2 polarization, accompanied by improved osteo-immunomodulatory behavior and superior bone regeneration in scaffold-treated groups compared with controls. This supports the concept that NP-reinforced matrices can bias macrophage fate through integrated physicochemical cues (e.g., viscoelasticity, nanoscale interfaces) while simultaneously supporting osteogenesis [121]. At the implant interface level, Zhu et al. provided mechanistic evidence that surface topography can regulate macrophage polarization in ways that translate to improved implant-to-bone integration. By systematically designing honeycomb-like micro/nanotopographies, they showed that immune responses at the interface could be shifted toward a more regenerative profile, ultimately facilitating osteointegration reinforcing that macrophage phenotype is “engineerable” via material cues [167]. Shayan et al. reported that nanopatterned bulk metallic glass surfaces modulated macrophage polarization, demonstrating that nanoscale patterning can be used to selectively tune macrophage inflammatory programs. While not a NP carrier system per se, these data strongly complement NP studies by showing that macrophage fate can be regulated through nanoscale biophysical signaling, which is directly relevant when designing NP coatings and nano-textured implant-adjacent interfaces [168]. Together, these studies substantiate that macrophage polarization can be guided by (i) NP size and composition (e.g., nanoHA), (ii) controlled delivery of immunoregulatory cues (e.g., IL-4), and (iii) nano-enabled matrix/interface design (NP-reinforced scaffolds; nanotopography). Critically, the shared outcome across platforms is not only reduced pro-inflammatory signaling but also improved coupling between angiogenesis and osteogenesis, consistent with a macrophage-mediated pro-regenerative osteoimmune niche.

### 4.2. T Cell and Dendritic Cell Interactions

**Figure 4:**
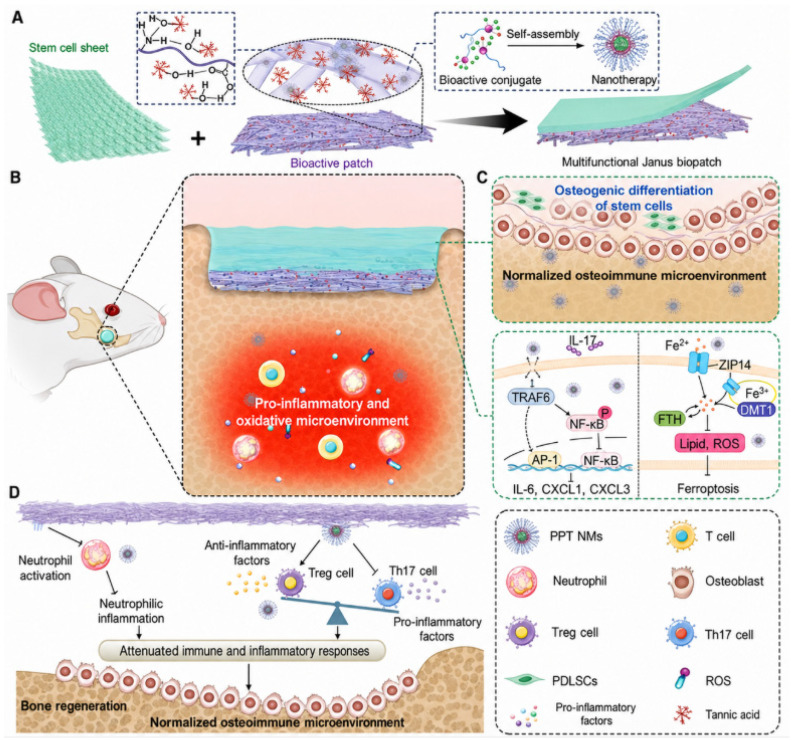
Schematic illustration of engineering a multifunctional Janus biopatch for bone regeneration. Reproduced with permission from [111]. Copyright 2024 Wiley (**A**) A sketch showing the structure, compositions, and development of the multifunctional Janus biopatch. The Janus biopatch consists of an inflammation-resolving membrane and a stem cell sheet derived from periodontal ligament stem cells (PDLSCs). In the bioactive membrane, an anti-inflammatory nanotherapy PPT NMs assembled by an amphiphilic conjugate is embedded in electrospun nanofibers, which are further functionalized with tannic acid (TA). (**B**) Schematic of the treatment of a bone defect with the Janus biopatch. The bioactive membrane is planted onto the interior side, while the cell sheet is located outside. (**C**) Cellular and molecular mechanisms underlying bone regeneration effects of PDLSCs in the Janus biopatch. The upper panel shows the gradual release of the nanotherapy concomitant with degradation of the fibrous membrane, thus affording an improved pathological microenvironment and facilitating differentiation of PDLSCs into osteoblasts in the bone defect. Meanwhile, degradation of the fibrous membrane enables infiltration of osteoblasts into the interior of the Janus biopatch to promote bone regeneration. The low panel illustrates signaling pathways underscoring bioactivities of PPT NMs in PDLSCs. (**D**) A diagram indicates the regulation of the osteoimmune (immune-inflammatory) microenvironment by PPT NMs to promote bone regeneration.

While macrophages dominate the early innate immune response during bone healing, adaptive immune cells, particularly T lymphocytes and dendritic cells, play a pivotal role in shaping long-term regenerative outcomes. These cells regulate immune memory, tolerance, and sustained inflammation, thereby influencing both bone formation and resorption processes. NPs provide a versatile platform to modulate adaptive immune interactions in a manner that favors bone regeneration. Dendritic cells function as professional antigen-presenting cells and act as a critical bridge between innate and adaptive immunity. Upon encountering foreign materials or damage-associated signals, dendritic cells undergo maturation and migrate to lymphoid tissues, where they activate naïve T cells. Excessive dendritic cell activation can lead to heightened adaptive immune responses and chronic inflammation, which are detrimental to bone healing. NP properties such as size, surface chemistry, and protein adsorption profiles strongly influence dendritic cell uptake and maturation. Several NP systems have been shown to induce a tolerogenic dendritic cell phenotype characterized by reduced expression of co-stimulatory molecules and increased secretion of immunoregulatory cytokines. This shift limits excessive T cell activation and promotes immune tolerance at the implantation site. By modulating dendritic cell behavior, NPs indirectly regulate downstream T cell responses and establish a balanced immune environment conducive to regeneration. T lymphocytes exert complex and context-dependent effects on bone remodeling. Pro-inflammatory T cell subsets can stimulate osteoclastogenesis through the secretion of cytokines such as tumor necrosis factor-α and interleukin-17, leading to bone resorption. In contrast, regulatory T cells suppress excessive immune activation and support osteoblast function. The balance between these T cell subsets is therefore a key determinant of regenerative success (Figure 4) [111]. NPs influence T cell responses both directly and indirectly. Direct interactions occur when NPs or their released cargo modulate T cell activation, proliferation, or differentiation. Indirect effects are mediated through upstream regulation of antigen-presenting cells and macrophages. For instance, NPs that promote a tolerogenic dendritic cell phenotype often result in increased regulatory T cell populations and reduced pro-inflammatory T cell activity. Targeted delivery strategies further enhance the precision of adaptive immune modulation. NPs functionalized with targeting ligands can preferentially accumulate in lymphoid tissues or interact with specific immune cell subsets. This selective engagement minimizes off-target immune effects while amplifying regenerative signaling pathways. Moreover, NPs capable of controlled or sequential release allow immune modulation to be synchronized with different stages of bone healing, from early inflammation to late-stage remodeling. The interplay between T cells, dendritic cells, and skeletal cells underscores the importance of viewing bone regeneration as an immunologically active process rather than a purely structural one. By regulating adaptive immune mechanisms, NP-based systems extend their influence beyond the local defect site and shape systemic immune responses that contribute to long-term bone homeostasis. Experimental evidence increasingly supports the view that NP design can be leveraged to reprogram dendritic cell–T cell crosstalk toward a pro-regenerative immune setpoint, thereby limiting chronic inflammation-driven osteolysis while preserving osteogenic coupling. A representative tolerance-oriented strategy was reported by Getts et al., who fabricated negatively charged, antigen-decorated microparticles (~500 nm) that were preferentially processed by antigen-presenting cells and induced durable T cell tolerance in vivo. Although developed in an autoimmune disease model, the work provides a strong mechanistic framework for biomaterial-associated settings: particle charge and APC handling were sufficient to suppress pathogenic T cell activation without broad immunosuppression—an outcome conceptually aligned with preventing maladaptive adaptive immunity (e.g., Th1/Th17 skewing) around regenerative implants [169]. Complementing this, Maldonado et al. developed PLGA NPs co-delivering antigen with rapamycin to drive tolerogenic dendritic cell programming (reduced co-stimulation) and promote regulatory T cell (Treg) responses. Their findings demonstrated that co-encapsulation of a tolerogenic cue within a degradable nanocarrier can bias DC output away from inflammatory priming and toward regulatory circuits—an approach that is highly translatable to bone regeneration contexts where excessive DC activation can perpetuate osteoclastogenic cytokine loops [170]. Notably, adaptive immune reprogramming has also been linked directly to bone loss prevention in craniofacial inflammatory disease. Elashiry et al. generated dendritic cell-derived exosomes enriched with TGF-β1 and IL-10 (so-called regulatory/tolerogenic cargo) and showed that these vesicles suppressed DC maturation, restrained Th17 effector induction, and enhanced Treg recruitment, ultimately reducing osteoclast-associated inflammatory mediators and inhibiting alveolar bone loss in vivo. This study provides a compelling proof-of-concept that DC-directed tolerogenic signaling can reshape the downstream T cell landscape in a way that is bone-protective—directly relevant to NP-enabled immunomodulation strategies for regeneration [171]. In parallel, biomaterials engineered to recruit specific DC subsets have shown that APC composition at the injury site can be actively tuned. Lokwani et al. reported that pro-regenerative extracellular-matrix-derived biomaterials expanded a BATF3-dependent DC population associated with self-tolerance-linked programs, illustrating that material cues can shape immunoregulatory DC phenotypes in vivo. While performed in a traumatic injury context outside bone, the mechanistic insight—selective enrichment of immunoregulatory DCs by pro-regenerative materials—supports analogous NP strategies aimed at reducing excessive adaptive activation during late-stage remodeling [172]. These adaptive immune shifts are particularly consequential because distinct T cell subsets exert opposing effects on bone remodeling. Sato et al. established that Th17 cells can function as potent osteoclastogenic helpers by driving osteoclastogenesis in inflammatory settings, providing a mechanistic link between T cell polarization and bone destruction pathways [173]. Conversely, regulatory T cells can act as a protective brake on osteoclast formation. Zaiss et al. reported that Treg cells suppress osteoclastogenesis, supporting the broader concept that strategies enriching Tregs (often downstream of tolerogenic DC phenotypes) can shift the immune–skeletal balance away from resorption and toward regeneration [174]. Taken together, these studies support a coherent design principle for bone-regenerative nanomedicine: NPs (and closely related micro/nanoparticulate systems) can be engineered to restrain DC maturation, promote tolerogenic antigen presentation, and bias T cell differentiation toward Treg-dominant rather than Th17/Th1-dominant responses (Table 4). Through this route, NP-mediated adaptive immune modulation can reduce osteoclastogenic signaling and sustain a permissive immune state that supports angiogenesis–osteogenesis coupling during late-stage remodeling.

**Table 4:** NP Effects on Dendritic Cells and T Cell Responses.

NP System	Target Immune Cells	Key DC/T-Cell Effect	Context	Reference
Dendritic cell-membrane-mimicking NPs (DCMNPs)	DCs & adaptive immune cells	Elicits humoral and cellular immune responses indicating DC uptake and downstream T-cell activation	Infectious immunotherapy model	[175]
Antigen-Capturing Dendritic-Cell-Targeting NPs (in situ vaccine)	DC antigen capture & T-cell priming	Enhances antigen presentation by DCs and improves antitumor T-cell responses	Cancer immunotherapy	[176]
Cell membrane-coated mRNA NPs	DCs → CD8^+^ T cells	DC-targeted mRNA delivery leads to robust antigen presentation & increased antigen-specific CD8^+^ T cells	Antitumor model	[177]
Engineered lipid NPs for DC targeting	DCs & antigen presentation	Mannose receptor-directed LNPs enhance DC uptake and antigen translation for T-cell activation	Cancer immunology	[178]
hr-8-PLGA@Ag/CpG nanovaccine	DCs & CD8^+^ T cells	DC-targeted PLGA NP increases antigen cross-presentation and CD8^+^ T-cell activation	Antitumor vaccine research	[179]
Lipid NPs for DC + mRNA delivery study	Dendritic cells & T responses	Engineered LNPs for potent adaptive immunity via DC processing	Immune engineering (vaccination)	[180]
Designer Self-Emulsifying Nanovaccines	DCs & T cells	Nanovaccine shows dual engagement of DC and T-cell pathways (more immune activation)	Vaccine immunology	[181]
Engineered Hybrid Membrane-Coated NPs for DC & T activation	DCs → CD4^+^/CD8^+^ T cells	Hybrid NPs activate DCs and promote T-cell recruitment/activation	Cancer immunotherapy	[182]
(Related Antigen-Targeting NP) DC-targeting nanovaccine enhancing CD8^+^ T cell responses	DCs & CD8^+^ T cells	Peptide-targeted DEC-205 delivery enhances DC maturation & CD8 T cell activation	Tumor immunotherapy model	[179]

## 5. Targeted Delivery and Temporal Control

Effective bone regeneration requires not only the presence of bioactive signals but also their precise spatial and temporal presentation within the defect microenvironment. Immune responses during bone healing are highly dynamic, evolving from an early inflammatory phase to subsequent stages of resolution, angiogenesis, osteogenesis, and remodeling. Disruption of this tightly regulated sequence often results in delayed healing or fibrotic outcomes. NP-based systems offer unique advantages for targeted delivery and temporal control of immunomodulatory and osteogenic cues, enabling synchronization of immune modulation with the natural progression of bone regeneration. Targeted delivery strategies aim to localize NPs at the bone defect site or direct them toward specific cell populations, thereby enhancing therapeutic efficacy while minimizing systemic side effects. Temporal control, on the other hand, ensures that immune modulation occurs at appropriate stages of healing, preventing premature suppression of inflammation or prolonged immune activation. Together, these design principles position NPs as dynamic regulators rather than static components of bone regenerative therapies.

**Figure 5:**
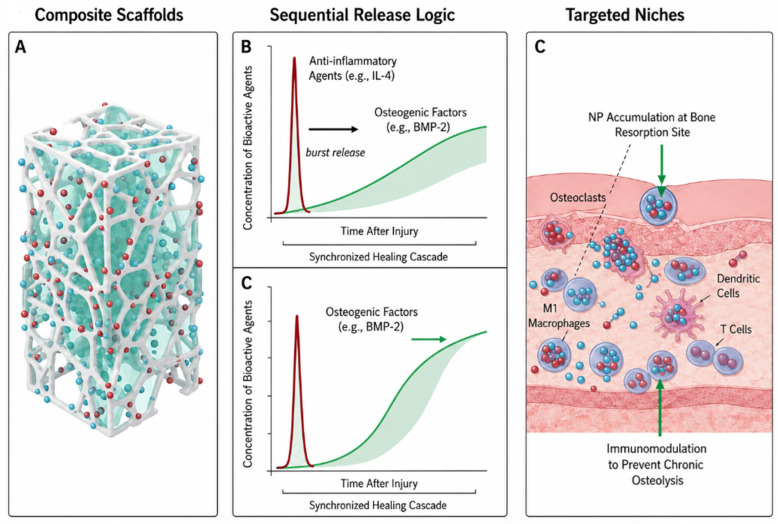
Schematic representation of spatiotemporal control strategies and scaffold integration for synchronized immune-guided bone regeneration. illustrates the integration of NPs into macro-scale constructs to achieve precise regenerative control. (**A**) Composite Scaffolds show the incorporation of NPs into 3D-printed lattices and hydrogels for mechanical stability and localized delivery. (**B**) Sequential Release Logic details the temporal transition from an early anti-inflammatory burst release (e.g., IL-4) to a sustained osteogenic phase (e.g., BMP-2) to synchronize the healing cascade. (**C**) Targeted Niches visualize the spatial localization of NPs at bone resorption sites and their interaction with Dendritic Cells (DCs) and T cells to modulate the immune environment and prevent osteolysis. Together, these strategies facilitate a coordinated transition from inflammation to structural bone regeneration. The figure was initially designed using BioRender and subsequently edited and finalized with Adobe Illustrator 2024. Dedecengiz Varol, G. (2026).

The orchestration of bone regeneration, as depicted in Figure 5, represents a synergistic approach that bridges macro-scale structural engineering with micro-scale immunomodulation. In Figure 5A (Composite Scaffolds), the integration of NPs into 3D-printed PCL/PLGA lattices and GelMA hydrogels creates a multifunctional platform that provides immediate mechanical stabilization while acting as a reservoir for localized therapeutic delivery. This prevents the rapid systemic clearance of bioactive agents, ensuring their sustained presence at the defect site. Figure 5B (Sequential Release Logic) highlights the critical importance of temporal signaling, where the system is engineered to provide an initial burst release of anti-inflammatory agents (e.g., IL-4) to resolve the acute inflammatory phase, followed by a controlled, long-term delivery of osteogenic factors (e.g., BMP-2) to facilitate matrix mineralization and structural maturation. Furthermore, Figure 5C (Targeted Niches) demonstrates the spatial precision of the platform, showing NP accumulation at bone resorption sites to counteract osteoclast-mediated bone loss and their direct interaction with Dendritic Cells (DCs) and T cells to shift the adaptive immune response toward a pro-regenerative state. Collectively, these integrated strategies transform bone regeneration from a passive process into a dynamic, immune-guided healing cascade.

### 5.1. Targeted Delivery to the Osteoimmune Microenvironment

The osteoimmune microenvironment is characterized by a heterogeneous population of immune cells, stromal cells, and vascular components, each contributing to regenerative outcomes. NP targeting can be achieved through passive or active mechanisms. Passive targeting relies on physicochemical properties such as particle size, shape, and surface charge, which influence NP retention within bone defects and uptake by immune cells. Due to their phagocytic nature, macrophages readily internalize NPs, making them a primary target for immunomodulatory interventions. Active targeting strategies involve surface functionalization with ligands that recognize specific receptors expressed by immune or osteogenic cells. Peptides derived from extracellular matrix proteins, carbohydrate moieties, and small molecules have been employed to enhance NP affinity for macrophages, dendritic cells, or mesenchymal stem cells. Such targeting improves cellular specificity and amplifies localized immune modulation without affecting systemic immune homeostasis. Bone-targeting approaches further refine spatial control by exploiting the mineralized nature of skeletal tissue. Functionalization with molecules that exhibit high affinity for calcium-rich environments allows NPs to preferentially accumulate in bone defects. This localization enhances the concentration of immunomodulatory agents at the site of regeneration, thereby reinforcing local immune–osteogenic coupling. Importantly, targeted delivery reduces the risk of off-target immune suppression, which could compromise host defense mechanisms. Several studies provide direct proof that bone-homing ligands can markedly increase NP accumulation in mineralized niches, thereby amplifying local immunomodulation while limiting systemic exposure. For instance, Ryu et al. conjugated alendronate (a bisphosphonate with high hydroxyapatite affinity) onto nanodiamond drug carriers to create a bone-targeted platform. They reported substantially enhanced hydroxyapatite binding and preferential cellular uptake in osteoblast-like cells compared with non-targeted nanodiamonds, supporting the concept that bisphosphonate functionalization can enrich nanocarriers at bone surfaces and improve local payload availability [183]. In parallel, aspartate-rich bone-targeting peptides have been widely used to exploit electrostatic affinity for calcium-rich minerals. Wang et al. performed pharmacokinetic and biodistribution studies on bone-targeted carriers using alendronate and D-Asp8 (D-aspartic acid octapeptide) as osteotropic ligands, showing that these moieties enable strong binding to hydroxyapatite and favor bone-associated distribution—establishing a quantitative foundation for ligand-guided bone localization [184]. Building on this principle, Low et al. designed a bone-targeted, acid-sensitive doxorubicin conjugate in which D-Asp8 served both as the bone-homing motif and as part of the carrier architecture, demonstrating that osteotropic peptides can be combined with stimulus-responsive chemistry to sharpen spatial control while limiting premature release [185]. Importantly, bone targeting can be refined even further by aiming at specific bone compartments that are immunologically active. Cai et al. developed a bone-resorption surface-targeting NP guided by Asp8, motivated by evidence that Asp8 preferentially binds highly crystallized hydroxyapatite found at active resorption interfaces. Their in vivo work supports the idea that osteotropic ligands can be selected not only for “bone vs. non-bone” discrimination, but also for preferential enrichment at disease- or remodeling-associated mineral surfaces—precisely where osteoimmune signaling is heightened [186]. Beyond mineral-directed targeting, immune-cell-biased NP uptake can be engineered through rational control of physicochemical parameters and ligand presentation—an approach particularly relevant for macrophage-centered osteoimmunomodulation. A landmark study by Getts et al. demonstrated that negatively charged poly(lactide-co-glycolide) (PLGA) microparticles selectively accumulated in antigen-presenting cells following systemic administration and induced antigen-specific immune tolerance. Their findings showed that particle surface charge critically influenced biodistribution and phagocytic processing, enabling immune reprogramming without global immunosuppression. This work established surface electrostatics as a powerful design parameter for directing NP–immune cell interactions in vivo [169]. Receptor-mediated targeting strategies have further refined immune specificity. Glass et al. developed mannose-functionalized polymeric micelles using click-chemistry conjugation to enhance selective recognition by macrophages expressing the mannose receptor (CD206). Their study demonstrated significantly increased uptake in human macrophages compared with non-functionalized controls, confirming that carbohydrate ligands can preferentially engage macrophage subsets associated with reparative and anti-inflammatory functions. This approach highlights how receptor-specific surface engineering can improve macrophage-directed delivery and enable more precise immunomodulatory interventions within regenerative microenvironments [187]. Targeted delivery can also be directed toward osteogenic niches to reinforce immune–osteogenic coupling. Low et al. designed a bone-targeted, acid-sensitive doxorubicin conjugate incorporating an Asp8 peptide (octa-aspartate) with strong affinity for hydroxyapatite. Their in vivo studies confirmed preferential accumulation in bone tissue and controlled release under acidic conditions, demonstrating that osteotropic ligands can be integrated with stimulus-responsive systems to sharpen spatial precision in skeletal applications [185].

### 5.2. Temporal Regulation of Immune Modulation

Temporal control is particularly critical in osteoimmunology, as inflammation plays both beneficial and detrimental roles depending on its timing and duration. Early inflammatory signaling is necessary for debris clearance, immune cell recruitment, and initiation of repair cascades. However, prolonged or excessive inflammation inhibits osteogenic differentiation and promotes osteoclast-mediated bone resorption. NP-based delivery systems enable fine-tuning of immune responses across different phases of bone healing. Controlled release mechanisms are commonly employed to achieve temporal regulation. Biodegradable polymeric NPs can be engineered to release encapsulated agents over predefined timeframes, ranging from rapid initial release to sustained delivery over weeks. Such systems allow early-phase support of inflammatory signaling followed by gradual transition toward anti-inflammatory and pro-regenerative cues. This staged release mirrors the physiological progression of bone regeneration and reduces the likelihood of immune dysregulation. Sequential delivery platforms represent an advanced strategy for temporal immune control. In these systems, NPs are designed to release distinct bioactive agents at different stages of degradation. For example, early release of immunostimulatory factors can support initial macrophage recruitment and activation, while later release of anti-inflammatory or osteogenic agents promotes tissue formation and remodeling. By coordinating immune modulation with osteogenesis, sequential delivery systems enhance the overall regenerative outcome. A number of experimental studies have confirmed that precisely timed immune modulation enhances bone regeneration and that NP and scaffold delivery platforms can be engineered to achieve such temporal control. For example, Spiller et al. showed that sequential presentation of inflammatory cues enhances vascularization in engineered tissues. In their work, scaffolds with early delivery of interferon-γ (IFN-γ) followed by interleukin-4 (IL-4) created a transient inflammatory phase followed by a reparative phase, which correlated with increased angiogenesis in a murine implantation model compared to scaffolds lacking staged cytokine presentation. This sequential strategy mirrors physiological healing, wherein an initial pro-inflammatory phase supports blood vessel ingrowth prior to tissue formation [188]. Controlled release systems have similarly been employed to modulate macrophage function over time. Yang et al. described a sequential release design in which IFN-γ and IL-4 were loaded into discrete layers of hydroxyapatite nanotube arrays on titanium, with the spatial arrangement and polymer carriers engineered to produce an early burst of IFN-γ followed by delayed IL-4 release. Their system was intended to time macrophage phenotype switching at specific stages, thereby regulating early inflammatory responses and promoting later osteogenic signaling at implant interfaces [96]. Temporal regulation can also be implemented through injectable, degradable micro-/nano-systems that reshape the osteoimmune milieu during defect healing. For example, Xu et al. fabricated injectable organic/inorganic microfluidic microspheres and showed that their material-driven immune effects were accompanied by macrophage polarization changes and a secretome favorable for repair, ultimately improving bone regeneration outcomes in vivo [189]. Beyond cytokines, sequential delivery has been extended to nucleic-acid timing to more tightly control phenotype transitions. Li et al. reported sequential delivery of distinct microRNA nanocarriers (miR-155 followed by miR-21) and demonstrated that the schedule itself enabled a more efficient M1-to-M2 transition aligned with the early inflammatory-to-reparative timeline of bone healing—highlighting that temporal “immune programming” can be achieved not only by what is delivered, but by when it is delivered [190]. More broadly, multi-factor architectures that intentionally decouple early and late biological functions are increasingly being validated in bone regeneration. For instance, Niu et al. developed an injectable hydrogel system enabling sequential PDGF-BB (angiogenic) then BMP-2 (osteogenic) delivery, showing that staged presentation improved vascularized bone regeneration compared with non-sequential designs—consistent with the requirement for early vascular support followed by sustained osteoinduction during remodeling [191].

### 5.3. Stimuli-Responsive NPs for Adaptive Control

Stimuli-responsive NPs introduce an additional layer of temporal precision by responding to endogenous signals within the bone microenvironment. Inflammatory bone defects are characterized by changes in pH, redox status, and enzymatic activity, which can be exploited as triggers for controlled release. NPs sensitive to acidic conditions can selectively release immunomodulatory agents in inflamed regions, thereby limiting their activity to pathological sites. Enzyme-responsive systems are particularly attractive for bone regeneration, as proteases and matrix-degrading enzymes are upregulated during inflammation and remodeling. NPs designed to degrade in response to these enzymes enable context-dependent release of therapeutic payloads, ensuring that immune modulation adapts to the evolving biological environment. This adaptive behavior minimizes unnecessary intervention and preserves physiological immune functions. Redox-responsive NPs further contribute to temporal control by releasing antioxidants or anti-inflammatory agents in response to elevated ROS levels. By mitigating oxidative stress, these systems protect osteogenic cells and promote a regenerative immune phenotype. Collectively, stimuli-responsive NPs offer a biomimetic approach to immune regulation that aligns therapeutic action with endogenous healing signals.

For instance, Martinez-Carmona et al. designed pH-responsive mesoporous silica NPs capped with an acid-labile polymer layer that remained stable at physiological pH but underwent accelerated cargo release under acidic conditions resembling inflammatory microenvironments. When loaded with therapeutic agents, the system demonstrated enhanced payload release specifically at pH levels typical of inflamed bone sites, suggesting strategic control of drug delivery in response to pathological stimuli [192]. In enzyme-responsive contexts, studies such as those reviewed by Sullivan et al. have developed matrix metalloproteinase (MMP)-sensitive micellar NPs incorporating protease-cleavable sequences, which undergo structural degradation and release encapsulated drugs in regions of elevated MMP activity. Elevated expression of MMPs—particularly MMP-2 and MMP-9—is characteristic of inflammatory and remodeling stages of bone healing, underscoring the utility of enzyme-triggered systems for context-dependent therapeutic release [104]. Although specific applications in bone regeneration remain a developing area, broader evidence from responsive biomaterials research indicates that Pi et al. highlight enzyme-responsive systems including NPs and hydrogels engineered to undergo payload release upon exposure to endogenous enzymes, demonstrating the feasibility of harnessing pathological enzymatic cues for localized therapy [193]. Collectively, these examples show that stimuli-responsive NPs can be engineered to remain inert under homeostatic conditions but trigger therapeutic release upon encountering specific microenvironmental signals such as reduced pH or elevated proteolytic enzyme levels, thereby aligning immunomodulatory action with intrinsic cues of inflammation and remodeling in bone defects.

### 5.4. Integration of Targeting and Temporal Control in Osteoimmunomodulation

The greatest potential of NP-based systems lies in the integration of targeting and temporal control within a single platform. Multifunctional NPs capable of selective localization and stage-specific release enable precise orchestration of immune responses throughout bone regeneration. Such systems move beyond conventional delivery paradigms and actively participate in shaping the immune microenvironment. By directing NPs toward key immune cell populations and synchronizing immune modulation with osteogenic progression, these platforms enhance crosstalk between immune cells, mesenchymal stem cells, and endothelial cells. This coordinated interaction supports angiogenesis–osteogenesis coupling and promotes long-term tissue integration. Importantly, integrated targeting and temporal control strategies reduce the need for high therapeutic doses and lower the risk of adverse immune reactions. As bone regeneration is a multiscale and time-dependent process, future NP designs are expected to increasingly emphasize adaptive and responsive behaviors (Table 5). The convergence of materials science, immunology, and bioengineering will enable the development of intelligent NP systems that not only deliver therapeutic agents but also dynamically regulate immune processes to optimize skeletal repair. Spiller et al. investigated whether staged immune modulation could enhance vascularization and subsequent bone healing. They aimed to test the hypothesis that sequentially guiding macrophage phenotype (early pro-inflammatory M1 then later pro- regenerative M2) would improve angiogenesis and tissue repair. To that end the authors fabricated tissue engineering scaffolds designed to present cues that promoted an early M1 response followed by M2-favoring signals, and they evaluated macrophage phenotype, vessel formation, and tissue ingrowth in vitro and in vivo. The study found that scaffolds that promoted a controlled M1 → M2 transition increased angiogenic sprouting and vascular maturation, and correlated with improved regenerative outcomes compared with non-programmed scaffolds, supporting the concept that temporally tuned immune modulation is beneficial for repair [194]. Jing et al. set out to combine bone-targeting and carrier functionality by decorating polymeric NPs with an alendronate (bisphosphonate) ligand so that the delivery system would preferentially accumulate at mineralized bone surfaces. The work produced alendronate-decorated PLGA/related NPs, characterized their hydroxyapatite affinity and cellular uptake, and tested drug loading/release and bioactivity in cell assays relevant to bone cells. They reported that ligand-decorated particles exhibited markedly higher binding to hydroxyapatite and preferential uptake by osteoblastic cell lines, while maintaining sustained release properties; these features demonstrated feasible spatial targeting to bone and a platform that could be later combined with responsive release mechanisms to achieve spatiotemporal control [98]. Zhang et al. developed an MMP-responsive matrix designed to release an immunomodulatory lipid (phosphatidylserine) on demand with the objective of synchronizing immunoregulation with the inflammatory/remodeling phases of bone regeneration. The authors synthesized a PEG-peptide-PS network in which peptide linkers are cleaved by matrix metalloproteinases upregulated during early inflammation; they evaluated macrophage phenotype shifts, osteogenic markers, and new bone formation in defect models. The platform produced on-demand release in MMP-rich environments, induced macrophage polarization toward an anti-inflammatory M2-like state, enhanced osteogenic differentiation in vitro, and improved bone regeneration in vivo—demonstrating that environment-activated, temporally controlled release can effectively couple immune modulation to regenerative processes [195]. Wongpinyochit et al. investigated enzyme-triggered degradation of silk fibroin NPs to establish an enzyme-responsive release mechanism; using proteolytic enzymes (e.g., protease XIV) in vitro they characterized degradation kinetics and demonstrated that enzymatic exposure accelerated cargo release, supporting the feasibility of protease-responsive NPs for context-dependent delivery in inflamed/remodeling tissues [196]. Cui et al. engineered mesoporous silica NPs capped with disulfide-linked PEG gatekeepers to achieve glutathione (GSH)-sensitive cargo release; they demonstrated that intracellular-level GSH cleaved the disulfide bonds to open pore caps and trigger rapid payload release, providing a robust redox-responsive mechanism that can be combined with targeting ligands for stage-specific delivery in pathological microenvironments [197].

**Table 5:** Stimuli-Responsive NP Systems for Immunomodulated Bone Regeneration.

System (NP/Composite)	Stimulus	Response/Release Logic	Immuno-Regulatory Angle	Bone Model/Indication	Reference
Gelatin NPs (BMP9) coated with metal-phenolic network (TA-Ce)	Acidic inflammatory microenvironment (pH)	pH-adaptive response enabling microenvironment-specific action	Regulates macrophage–osteoclast axis; controls osteoclast fate in osteomyelitis	Osteomyelitis-related bone regeneration	[160]
HAp nanocomposite (HPDD) in hydrogel for controlled release	NIR + pH	Spatiotemporal release under NIR/pH stimulation	Reprograms anti-inflammatory immune microenvironment (via macrophage polarization + ROS clearance)	Bone defect repair	[198]
MOF- NP hydrogel coating on LCFRPEEK implant (HAP@Mg-GA MOF + methacryloyl chitosan)	Low pH (immune environment)	pH-sensitive biomolecule release; ROS/NO modulation in “immune” pH	“Immunomodulation” + reduced oxidative/inflammatory signals reported	Rabbit tibia defect + osseointegration	[199]
4D-printed scaffold with Fe_3_O_4_@SiO_2_ magnetic NPs (PLGA/HA composite)	NIR + static magnetic field	Shape change/recovery + navigation and therapy under dual stimuli	Pro-regenerative microenvironment via enhanced vascularized bone formation	Critical-sized irregular bone defects	[200]
3D-printed NIR-responsive shape-memory polyurethane/magnesium scaffold	NIR (photothermal trigger)	Tight contact + shape memory activation under NIR	Immune interface discussed via improved healing microenvironment	Robust bone regeneration	[201]
NIR-activable thermosensitive hydrogel “gate” on mesoporous bioactive glass scaffold (PTHrP-2 delivery)	On/Off NIR	Reversible phase transition enables pulsatile vs sustained dual-mode release	Indirect immuno-coupling via controlled remodeling signals (angiogenesis/osteogenesis axis)	Rat critical-size femoral defect	[202]
Supramolecular, nano-assisted dynamic hydrogel (polyphenol/polypeptide/clay nanosheets)	Dynamic (stimuli-adaptive supramolecular network)	Self-healing + adhesion + bioactive ion release	Explicit “immunomodulatory” activity reported; regulates inflammatory microenvironment	Irregular bone defect repair (in vivo)	[26]
Gold nanorods + nanohydroxyapatite hybrid hydrogel	NIR photothermal	Photothermal tumor clearance + regenerative hydrogel function	Post-operative microenvironment control (anti-tumor + repair)	Tibia osteosarcoma defect model	[203]
ROS-responsive hydrogel platform for aged bone regeneration (drug-loaded; ROS-triggered release)	ROS	ROS-sensitive responsiveness for on-site release	Osteoimmune microenvironment targeting (aged/inflammatory niche)	Aged bone regeneration	[204]
Photothermal-immunomodulatory nanohydrogel for infected bone defects	Photothermal (NIR)	Light-triggered antibacterial + pro-regenerative regulation	Explicit “immunomodulatory” design in infected bone defects	Infected bone defect regeneration	[205]

## 6. Preclinical Outcomes

Preclinical investigations provide critical evidence for the therapeutic potential of NP-based immunomodulatory strategies in bone regeneration. In vivo studies in small and large animal models have demonstrated that NP systems not only enhance new bone formation but also actively modulate local immune responses, particularly through macrophage phenotype switching, regulated cytokine profiles, and improved angiogenesis.

### 6.1. NP Effects on Bone Healing in Animal Models

One of the most frequently studied systems in preclinical bone regeneration research is mesoporous bioactive glass NPs. These mesostructured bioactive glasses have been evaluated for their capability to enhance bone regeneration while also interacting favorably with the host immune response. For example, mesoporous bioactive glass NPs implanted in bone defect models have been shown to modulate local macrophage behavior without triggering sustained pro-inflammatory M1 polarization, an outcome associated with improved tissue integration and regenerative progression. In particular, Feito et al. conducted comparative studies demonstrating that both bulk and nano-sized mesoporous bioactive glasses do not induce adverse M1 macrophage activation, suggesting that these NPs reside within the defect environment without provoking chronic inflammatory states that would otherwise interfere with healing processes [206]. Beyond intrinsic immunomodulation, mesoporous bioactive glass NPs provide controlled release of bioactive ions such as calcium and silica, which have been implicated in promoting osteogenic differentiation and supporting angiogenesis. Preclinical work indicates that these ionic dissolution products can enhance osteoblast recruitment and matrix deposition, while also reducing the expression of pro-inflammatory cytokines in defect sites. Collectively, these multifunctional behaviors position mesoporous bioactive glass NPs as strong candidates for osteoimmunomodulatory bone regeneration strategies. Similar promising outcomes have been reported for NP systems incorporating mineralized components such as hydroxyapatite. Hydroxyapatite NPs, due to their compositional similarity to the native mineral phase of bone, are widely used as osteoconductive agents in animal fracture and defect models. While much of the evidence on hydroxyapatite NPs focuses on their osteogenic potential, in vivo biodistribution and safety studies also support their biocompatibility and minimal systemic toxicity when locally delivered. For instance, an in vivo radio-labeled biodistribution study demonstrated that implanted hydroxyapatite particles remain localized within the bone defect site over long durations without inducing pathological changes in major organs, confirming their suitability for localized regenerative applications (e.g., in rat tibia) [207]. Furthermore, hydroxyapatite nanocomposites integrated within polymeric matrices have shown enhanced bone regeneration in preclinical settings, with improved new bone volume and mechanical strength compared with controls, highlighting the translational promise of mineral-based NPs for bone healing [40]. Bioactive glass NPs derived from classical 45S5 compositions have also undergone evaluation in vivo. For example, Nogueira et al. investigated the biological behavior of 45S5-based bioactive glass (SinGlass 45S5) and a modified high-potency variant (SinGlass High F18) in critical calvarial defect models in rats. Both materials promoted new bone formation and integration with surrounding bone tissue, with the high-potency variant displaying more rapid resorption and higher rates of bone maturation over a 42-day period, as evidenced by histomorphometric and micro-computed tomography analyses [208]. These findings underscore the osteogenic and immunocompatible behavior of 45S5-derived NPs in preclinical bone regeneration scenarios. For example, Zhao et al. investigated the use of three-dimensional printed strontium-containing mesoporous bioactive glass (Sr-MBG) scaffolds in a rat critical-sized calvarial defect model with the objective of harnessing ionic release to enhance bone regeneration. In this study, Sr-doped MBG scaffolds demonstrated high porosity and interconnected architecture that supported robust new bone formation and stimulated angiogenesis over eight weeks relative to undoped controls. Histological and micro-computed tomography analyses confirmed significant bone ingrowth with minimal inflammatory infiltration, indicating that Sr incorporation improved both osteoconduction and biocompatibility in vivo [209]. In another study, Wu et al. developed strontium-incorporated amino-functional MBG scaffolds and applied them in an osteoporotic rat model to assess both osteogenic and angiogenic stimulation in vivo. The Sr-modified scaffolds enhanced vascularization and new bone formation compared with non-modified matrices, and mechanistic analysis suggested suppression of oxidative stress and upregulation of pro-regenerative signaling pathways in bone marrow mesenchymal stem cells. These results highlight how trace element incorporation into NP scaffolds can augment both structural regeneration and host tissue compatibility in vivo [210].

Collectively, these in vivo studies highlight the ability of NP systems to support bone regeneration through both structural osteoconduction and favorable immune interactions. While precisely dissecting immunomodulatory mechanisms in animal models is complex, the observed outcomes indicate that NP compositions mimicking native bone mineral phases are capable of facilitating new bone tissue formation without prolonged inflammatory disruption. Such preclinical evidence strengthens the translational rationale for further development and optimization of NP-enabled therapies for bone defect repair.

### 6.2. Immunomodulation Linked to Bone Formation

Preclinical evidence increasingly supports the view that immune regulation is not merely a parallel phenomenon during bone regeneration, but a mechanistic driver of regeneration outcomes. In particular, biomaterial- and NP-mediated tuning of macrophage phenotype has repeatedly been associated with improved osteogenesis, enhanced vascular infiltration, and more organized remodeling. Excessive or persistent M1-like activation (often characterized by iNOS, TNF-α, IL-6, and sustained NF-κB signaling) can prolong the inflammatory phase and impair osteoprogenitor function. Conversely, timely emergence of M2-like macrophages (e.g., CD206, Arg1, IL-10, TGF-β signatures) supports resolution of inflammation, matrix deposition, and angiogenesis–osteogenesis coupling, which is essential for defect bridging and maturation. A clear illustration of how NP-enabled immune tuning can translate into improved bone formation comes from inorganic nanomaterials engineered to regulate oxidative stress and inflammatory signaling at the defect site. For example, CeO_2_ NPs (nanoceria) have been shown to influence macrophage polarization and concurrently promote osteogenic outcomes, leveraging their redox-active surface chemistry and ROS-scavenging capacity. In one representative study, valence-state manipulation of CeO_2_ NPs was linked to altered macrophage responses and improved osteogenic behavior, highlighting that immunomodulation and osteogenesis can be co-regulated by precise nanomaterial design rather than by drug payload alone [211]. In practical preclinical terms, this class of approach is relevant because oxidative stress and inflammatory persistence are common barriers in compromised healing (e.g., diabetes, infection-prone defects), and “immuno-redox” control provides a plausible route to restore a pro-regenerative trajectory. Beyond nanoceria, bioactive glass-derived nano/submicro systems provide another strong preclinical foundation for immune-linked bone formation. A seminal example is strontium-substituted bioactive glass engineered explicitly as an osteoimmunomodulatory material. In vivo and mechanistic work has shown that such bioactive glass systems can shape macrophage behavior while supporting downstream bone regeneration, consistent with the idea that ionic cues (e.g., Sr-containing dissolution products) participate in immune phenotype regulation and osteogenic stimulation simultaneously [212]. This matters for translational design because it provides a non-protein, non-gene approach to immune modulation—potentially improving stability, manufacturability, and regulatory tractability compared with growth-factor-heavy strategies. Importantly, the immune-to-bone coupling observed in these studies is frequently mirrored by changes in angiogenic and osteogenic mediators within defect sites. Even when BMP-2 is not delivered exogenously, immunomodulatory biomaterials can lead to elevated local expression of osteogenic and pro-vascular signals (commonly reported markers include BMP-2-related pathways, VEGF, and enhanced endothelial invasion), supporting the concept that immune reprogramming “unlocks” endogenous regeneration programs. Mechanistically, this coupling is consistent with broader osteoimmunology literature emphasizing macrophage-mediated coordination of vascular ingrowth and osteoprogenitor recruitment during the transition from inflammation to repair [213]. Crucially, preclinical datasets also indicate that NPs can influence adaptive immune cascades that indirectly shape bone formation quality—especially in conditions where immune imbalance drives bone loss or suppresses osteogenesis. A compelling in vivo example is the development of T cell-depleting NPs built on a mesoporous silica core and an immunologically active surface corona designed to eliminate activated T cells and restore immune homeostasis. In an ovariectomy (OVX) mouse model, this strategy improved bone-related outcomes by correcting pathological immune activation and normalizing downstream inflammatory tone, illustrating that NP immunotherapy can shift systemic/locoregional immune balance in ways that are favorable for bone formation [214]. While this model focuses on osteopenia rather than a focal defect, it provides high-quality preclinical evidence that NP control of adaptive immunity can rescue osteogenic deficiency—an insight directly relevant to bone-regenerative contexts where chronic inflammation and maladaptive T cell responses impair repair. With respect to dendritic cells, the preclinical literature increasingly frames them as a control node for determining whether biomaterial exposure leads to persistent immune activation or a more tolerogenic environment supportive of tissue repair. Although many dendritic-cell-focused studies are not exclusively “bone defect NP” papers, authoritative work has clarified how dendritic cell phenotype (mature immunogenic vs. tolerogenic) governs downstream T cell activation patterns and the chronicity of inflammation—parameters that strongly impact bone healing trajectories [215]. For bone regeneration-oriented reviews and design frameworks, this is typically discussed as: NP surface chemistry and protein corona → dendritic cell maturation state → T cell polarization (e.g., Th17/Treg balance) → osteoclast/osteoblast coupling and remodeling quality. Taken together, the preclinical evidence base supports a coherent mechanistic narrative: NPs can steer early innate immune behavior (especially macrophage polarization and inflammatory resolution) and, in selected contexts, reshape adaptive immune tone (T cells and dendritic-cell-mediated pathways). These changes correlate with improved angiogenesis, increased new bone volume, and enhanced structural maturation compared with non-immunomodulatory controls—supporting immune-informed NP design as a central strategy for enhanced bone regeneration rather than a secondary optimization. For example, Zhang et al. prepared strontium-substituted submicrometer bioactive glass (Sr-SBG) NPs and implanted them in murine defect models to evaluate whether ionic modulation of the immune microenvironment could enhance bone regeneration. The aim of the study was to determine if Sr incorporation into bioactive glass could modulate macrophage responses and simultaneously improve osteogenesis. Histological and in vivo assessments showed that Sr-SBG initiated a more favorable immune milieu with reduced pro-inflammatory signaling and enhanced osteoblast activity, resulting in significantly greater new bone formation compared with non-substituted bioactive glass controls. These findings support the concept that ionic cues released from nanoparticulate biomaterials can coordinate immune modulation and bone regeneration in vivo [212]. In another study examining the regenerative capabilities of cerium-containing NPs, Ren et al. demonstrated that nanoceria-loaded nanofibrous membranes significantly enhanced bone regeneration in periodontal defect models. The objective was to determine whether CeO_2_ NPs could exert antioxidative and immunomodulatory effects that support osteogenesis when incorporated into a biodegradable matrix. Micro-computed tomography and histopathological evaluation revealed accelerated new bone formation and improved mineralization in membranes containing CeO_2_ NPs compared with control scaffolds, suggesting that ceria’s redox biology may enhance bone formation when appropriately integrated into a nanostructured biomaterial [216]. Li et al. investigated the effects of valence-state-controlled CeO_2_ NPs incorporated onto titanium implant surfaces to determine whether tuning redox activity could modulate macrophage polarization and improve new bone formation. The purpose of the study was to assess how increased Ce^4+^/Ce^3+^ ratios on CeO_2_ NP surfaces influence immune response and osseointegration in vivo. In a rat femoral implantation model, surfaces with a higher Ce^4+^/Ce^3+^ ratio promoted macrophage transition toward an anti-inflammatory phenotype and enhanced new bone formation adjacent to the implant compared with unmodified titanium, demonstrating that redox-active NP design can directly regulate immune behavior and stimulate osteogenesis [211]. In addition, Shan et al. examined the incorporation of CeO_2_ into zirconia toughened bioceramic scaffolds in a rat bone defect model with the aim of achieving both antioxidative immunomodulation and accelerated osteogenesis. Their results showed that CeO_2_ incorporation promoted new bone formation and improved bone–implant integration compared with scaffolds lacking ceria, providing in vivo evidence that cerium-based nanomaterials can enhance skeletal repair through immune and oxidative stress regulation [217].

### 6.3. Integration with Scaffold Systems and Composite Implants

NPs are frequently integrated into 3D scaffolds and composite implants to address two interdependent requirements of bone regeneration: (i) mechanical/architectural support that maintains defect space and guides tissue ingrowth, and (ii) spatiotemporally controlled delivery of osteogenic and immunomodulatory cues that shape the local healing trajectory. This integration is especially important in immune-active bone defects, where the early inflammatory milieu can derail osteogenesis if not properly resolved. Embedding NPs within hydrogels, electrospun matrices, or 3D-printed polymer frameworks improves their local retention, reduces burst loss, and enables dose-efficient, localized immunomodulation—often yielding superior outcomes compared to NPs injected alone.

#### 6.3.1. Composite Hydrogel—NP Constructs

Injectable and moldable hydrogels (e.g., GelMA-based systems) are particularly attractive carriers because they can fill irregular defects, support cell infiltration, and serve as a depot for NP-mediated release. In animal models, composite hydrogel—NP constructs commonly demonstrate improved recruitment of osteoprogenitors and enhanced vascular infiltration—two processes strongly linked to immune resolution and M2-skewed macrophage signaling. A representative in vivo example is an injectable GelMA-based nanocomposite hydrogel (TPQGel) designed to reprogram macrophages from M1 to M2, promote vascularization, and accelerate bone defect repair. In that study, the hydrogel’s pH-responsive behavior supported phase-appropriate release, with clear evidence of reduced M1-associated markers (e.g., CD86) and increased M2-associated markers (e.g., CD206), accompanied by enhanced angiogenic signaling (CD31/VEGF) and more complete defect bridging in rats over a short healing period [218]. Mechanistically, these composite systems work through multiple layers: (1) material-driven immune tuning (surface chemistry and ionic cues that affect macrophage phenotype), (2) controlled release of peptides/small molecules from NPs (reducing prolonged inflammatory signaling), and (3) microstructural guidance provided by the hydrogel network that supports cell migration and matrix deposition. Notably, immunomodulatory effects observed in vivo are often paralleled by increased expression of osteogenic markers (e.g., RUNX2/OPN/BMP-2 axis) within the defect region, suggesting that immune reprogramming is functionally coupled to osteogenic progression rather than being a secondary, incidental outcome [218]. A second, highly relevant scaffold–NP strategy is the reinforcement of hydrogels with nanofibrous elements and bioactive NPs to improve both mechanics and bioactivity. For example, a composite system combining GelMA with coaxial nanofibers and bioactive glass was reported to improve angiogenesis and osteogenic marker expression and to enhance bone regeneration in a critical-size cranial defect setting [218]. While the osteogenic benefit is often emphasized, these systems are also increasingly discussed within an osteoimmunology framework because bioactive glass dissolution products (e.g., calcium/silicate ions) and nanoscale topographies can shift inflammatory tone and support a more pro-regenerative immune environment. For example, Yu et al. developed a GelMA hydrogel reinforced with polycaprolactone-coated GelMA nanofibers and bioactive glass NPs to evaluate whether multi-scale structural cues could enhance bone regeneration in vivo. The aim of the study was to combine nanoscale bioactivity with hydrogel mechanical reinforcement to support vascular infiltration and osteogenic progression. In a critical-size cranial defect model, the composite hydrogel significantly improved new bone formation and defect bridging compared with hydrogel alone. These outcomes were associated with enhanced angiogenesis and favorable local tissue responses, demonstrating that nanofiber/bioglass-augmented hydrogels can synergistically modulate both mechanical and biological aspects of bone regeneration [219]. Similarly, Zhang et al. reported the development of an injectable multifunctional nanocomposite GelMA hydrogel (TPQGel) designed to release bioactive peptides in response to local pH changes in a bone defect. The purpose of the study was to achieve phase-appropriate release of immunomodulatory and angiogenic cues and thereby reprogram macrophages and accelerate healing. In vivo results showed that TPQGel hydrogel promoted macrophage polarization toward an anti-inflammatory phenotype, increased expression of vascular growth markers (CD31/VEGF), and enhanced new bone formation in rat defect sites compared with non-responsive controls. The authors concluded that coupling pH-responsive NP release with a supportive hydrogel matrix effectively enhances bone regeneration by coordinating immune modulation with angiogenesis [218]. In addition, Lv et al. investigated a composite hydrogel in which a zinc-containing bioactive glass NP was combined with a gelatin-based hydrogel to determine whether the integrated system could increase angiogenesis and bone formation in vivo. The aim was to leverage the osteogenic and pro-angiogenic effects of zinc along with hydrogel structural support. Their findings demonstrated that the composite hydrogel markedly accelerated bone regeneration and vascular infiltration in rat femoral defects relative to hydrogel alone, indicating that metal-doped bioactive glass NPs embedded in hydrogel networks can promote multiple regenerative processes concurrently [220]. Yu et al. examined a Sr-CSH (strontium-calcium sulfate hemihydrate) NP–GelMA composite hydrogel with the objective of promoting in situ bone regeneration by combining sustained ionic release with injectable hydrogel support. In vivo experiments indicated that this composite significantly enhanced new bone formation in defect sites compared with hydrogel alone, with histological evidence of mature bone tissue development. Their results underscored how integration of ionic nanofillers into injectable hydrogels can provide both mechanical support and osteopromotive signaling in regenerative contexts [221].

#### 6.3.2. NPs Combined with Polymeric Carriers and 3D-Printed Scaffolds

Beyond hydrogels, NPs are widely incorporated into polymeric scaffolds (PCL, PLGA, etc.) and 3D-printed constructs to couple load-bearing architecture with immunomodulatory release. This composite approach is particularly important for defects requiring mechanical stability, where a purely hydrogel-based implant may be insufficient. In such designs, NPs can either be physically embedded within pores/fibers or chemically immobilized to enable sustained, localized release at the scaffold–tissue interface—precisely where inflammatory foreign body responses and fibrotic encapsulation are most likely to impair integration. A strong example of immunomodulation by release from a scaffold is the study “Sustained Release of Dexamethasone from 3D-Printed Scaffolds Modulates Macrophage Activation and Enhances Osteogenic Differentiation”. The authors showed that low-dose, localized dexamethasone release from 3D-printed scaffolds significantly enhanced macrophage phenotype switching toward a reparative profile and improved osteogenic differentiation outcomes—highlighting the value of scaffold-based delivery to coordinate macrophage–MSC crosstalk and reduce harmful persistence of inflammatory activation [222]. These findings align with a broader pattern in preclinical composite-implant studies: sustained local immunomodulation tends to correlate with (i) reduced chronic inflammatory signaling at the interface, (ii) improved vascular ingrowth, (iii) enhanced bone volume fraction, and (iv) better structural maturation/organization. In practical terms, NPs help convert scaffolds from passive supports into immune-instructive implants, capable of actively steering healing kinetics. When NP delivery is targeted and sustained, several studies report reductions in fibrotic tendencies and more favorable remodeling progression compared with conventional (non-controlled) delivery approaches—an effect that is typically attributed to early immune resolution and improved pro-regenerative cytokine profiles. For example, Majrashi et al. investigated whether low-dose, sustained dexamethasone release from 3D-printed PCL scaffolds could immunomodulate macrophage behavior and thereby enhance osteogenic differentiation. The purpose of the study was to determine if scaffold-based local release could promote reparative macrophage polarization and improve macrophage–MSC crosstalk without systemic exposure. They found that controlled dexamethasone release over several weeks markedly increased M1 → M2 phenotype switching in macrophages and enhanced late-stage osteogenic mineralization outcomes in MSC co-cultures, supporting the concept that 3D-printed scaffolds can serve as immune-instructive drug-release depots to improve osteogenesis [222]. Similarly, Li et al. studied 3D-printed PCL/PEG/hydroxyapatite composite scaffolds to evaluate how a structural parameter (pore size) shapes the foreign body response and macrophage polarization, and how these immune effects translate into bone regeneration. The aim was to identify pore architectures that reduce fibrotic tendencies while enhancing vascular and bone ingrowth. They found that pore size significantly altered the macrophage profile and FBR intensity, and that the pore design associated with a more pro-regenerative macrophage signature also produced greater vascular ingrowth and improved new bone formation in vivo, indicating that scaffold microarchitecture can be leveraged as an “immune design variable” in polymer—NP composites [223]. In another representative composite-implant strategy, Long et al. fabricated 3D-printed PLGA scaffolds incorporating black phosphorus (BP) with the objective of regulating the osteoimmune microenvironment and accelerating defect repair. The authors aimed to test whether BP-containing scaffolds could bias macrophage behavior toward a reparative phenotype while supporting osteogenic progression. They reported that the PLGA/BP scaffold promoted macrophage M2 polarization and improved bone regeneration performance relative to controls, supporting a model in which nanomaterial-enabled immune reprogramming and osteogenesis are coupled in 3D-printed polymer scaffolds [13]. Extending scaffold-based immunomodulatory release to NP carriers, Cong et al. developed an extrusion-printed chitosan scaffold containing dexamethasone-loaded mesoporous silica NPs (MSN@DEX) to combine mechanical reinforcement with sustained osteoinductive signaling. The purpose of the study was to determine whether NP-mediated dexamethasone delivery within a printable polymer matrix could enhance osteogenic differentiation while maintaining a clinically practical scaffold architecture. They found that incorporation of MSN@DEX produced a porous construct with improved mechanical performance and a release profile compatible with the timescale of bone healing, while significantly enhancing osteogenic differentiation readouts (e.g., ALP activity, mineral deposition, and osteogenic gene/protein expression) versus non- NP controls [224]. Collectively, these studies support the concept that polymeric/3D-printed scaffolds functionalized with NPs or nanofillers can provide mechanically stable architectures while enabling localized, sustained immunomodulation at the scaffold–tissue interface. This integrated approach tends to mitigate persistent inflammatory signaling, promote a pro-regenerative macrophage profile, and ultimately improve osteogenic outcomes, consistent with scaffold-enabled coordination of immune resolution and bone formation.

#### 6.3.3. Why Composites Often Outperform “NPs Alone” In Vivo

From a translational viewpoint, scaffold integration solves several recurring preclinical limitations: NP washout, burst release, poor defect retention, and lack of mechanical stability. Composite implants can also support multi-stage signaling, where early-phase immune tuning (e.g., limiting prolonged M1 activation) is followed by pro-osteogenic/angiogenic support in later stages. Hydrogels and printed scaffolds provide tunable microenvironments (porosity, stiffness, degradation) that synergize with NP cues. As a result, the most consistent preclinical success stories are increasingly those where NPs are deployed as part of a composite, defect-adapted construct rather than as standalone injections. Majrashi et al. aimed to determine whether scaffold-based, sustained immunomodulator release can outperform “drug/NP exposure alone” by preventing burst loss and maintaining a bioactive concentration at the cell–material interface. Using 3D-printed PCL scaffolds engineered for 35-day dexamethasone release, they found that scaffold-mediated delivery drastically enhanced M1 → M2 phenotype switching in macrophages and promoted osteogenic outcomes in macrophage–MSC co-culture (including mineralization dominated at later stages by local dexamethasone availability). These results support the concept that retentive, slow-release composites can more reliably coordinate immune resolution and osteogenesis than short-lived exposure conditions [222]. Song et al. aimed to test whether 3D-printed HA/PCL composite scaffold microarchitecture—a design element unavailable to “NPs alone”—can bias the immune microenvironment toward a pro-regenerative trajectory. By printing HA/PCL scaffolds with different fiber orientations, they found that the multi-angle interlaced (0-90-45) architecture most strongly promoted macrophage polarization toward an M2 phenotype, enhanced osteogenic differentiation of BMSCs, and upregulated angiogenic gene expression (e.g., VEGF/PDGF), indicating that structural control within composites can couple immunomodulation with angiogenesis–osteogenesis more effectively than non-architected delivery approaches [88]. Yang et al. aimed to address a clinically relevant scenario where “particles alone” frequently underperform—infected bone defects—by integrating antibacterial functionality and osteoconductive support into a 3D-printed PLGA/HA composite scaffold (with an antimicrobial surface component). They found that the multifunctional printed composite could inhibit bacterial biofilm formation while simultaneously promoting bone regeneration in vivo, illustrating how composite constructs can overcome simultaneous barriers (infection + inflammation + mechanical needs) that are difficult to solve with standalone NP administration [225]. Yang et al. further aimed to determine whether embedding peptide-loaded mesoporous silica NPs inside a hydrogel-infused 3D-printed PCL scaffold could provide defect-relevant mechanics while enabling controlled local release to support angiogenesis and osteogenesis. They reported that the resulting PM@GS/PCL composite scaffold exhibited robust osteogenic differentiation capacity in vitro and enhanced bone regeneration performance in a rat femoral defect model by micro-CT and histology, consistent with the premise that composite retention + controlled release yields stronger in vivo regeneration than non-retained delivery [226].

### 6.4. Limitations and Translational Challenges in Preclinical Studies

Despite the increasing number of preclinical studies demonstrating the promise of NP-enabled immunomodulation for bone regeneration, several limitations continue to hinder reliable clinical translation. One of the most fundamental challenges arises from intrinsic differences between animal and human immune systems. Rodent models, which dominate preclinical research, exhibit distinct macrophage activation profiles, cytokine signaling networks, and inflammatory resolution kinetics compared with humans, potentially leading to overestimation of immunomodulatory efficacy when results are extrapolated to clinical scenarios [227,228]. Another major limitation stems from the heterogeneity of preclinical bone defect models. Studies employ calvarial, femoral, tibial, and mandibular defects of varying sizes and healing capacities, each characterized by different mechanical loading conditions, vascularization patterns, and immune microenvironments. This variability complicates cross-study comparison and limits the establishment of standardized benchmarks for regenerative success [229]. Moreover, many investigations emphasize short-term endpoints such as early immune modulation and initial bone formation, while long-term remodeling, mechanical competence, and functional integration of regenerated bone tissue remain insufficiently evaluated [230]. Material-related factors further complicate translation. Although NPs can be precisely engineered under laboratory conditions, maintaining batch-to-batch reproducibility and physicochemical consistency during scale-up remains challenging. Minor variations in particle size distribution, surface chemistry, or degradation behavior can significantly influence immune cell interactions and downstream biological responses in vivo [231]. In addition, long-term biodistribution, degradation, and clearance of NPs are often inadequately characterized, raising concerns regarding accumulation in off-target organs, chronic inflammation, or delayed toxicity following repeated exposure [232]. A critical unresolved issue across the field is the lack of standardized quantitative parameters governing immunomodulation. While many NP systems demonstrate clear effects on macrophage polarization or cytokine expression in vivo, optimal dosing regimens, release kinetics, and therapeutic windows remain poorly defined. Immune modulation during bone healing is highly time-dependent; premature suppression of inflammation can impair early regenerative signaling, whereas prolonged modulation may disrupt immune homeostasis and hinder tissue remodeling [233]. Targeting specificity also represents a major translational challenge. Although macrophages readily internalize NPs, selective modulation of specific immune cell subsets without off-target effects remains difficult. NPs designed to influence macrophage polarization may also interact with dendritic cells or lymphocytes, potentially altering antigen presentation or adaptive immune responses in unintended ways, particularly in complex clinical settings involving infection or immune dysregulation [234]. Finally, regulatory and manufacturing barriers pose additional obstacles to clinical implementation. Multifunctional NP-based systems that combine structural scaffolds with immunomodulatory or stimuli-responsive components often fall into complex regulatory categories. The absence of clearly defined regulatory pathways, combined with stringent requirements for long-term safety and large-animal validation studies, continues to slow clinical translation [235]. Collectively, these limitations highlight the need for more standardized, mechanistically informed, and clinically relevant preclinical investigations. Greater emphasis on immune-specific endpoints, long-term functional assessment, scalable manufacturing strategies, and comparative large-animal models will be essential to bridge the gap between experimental success and clinical application.

## 7. Knowledge Gaps, Translational Challenges, and Future Perspectives

Future progress in NP-enabled modulation of the bone immune microenvironment will require a transition from proof-of-concept demonstrations toward mechanistically precise, clinically adaptable, and translationally viable systems. Although numerous preclinical studies have established the feasibility of immunomodulatory NP platforms, a deeper understanding of immune heterogeneity during bone regeneration remains essential. A major limitation of the current literature is the persistent reliance on oversimplified immune classifications, particularly the binary M1/M2 macrophage framework, which does not adequately capture the functional diversity and temporal plasticity of immune populations during bone repair. In addition, substantial variability in experimental models, immune profiling methodologies, and outcome measures hampers cross-study comparisons and limits the establishment of universally applicable design principles. Emerging single-cell transcriptomic and spatial proteomic approaches are expected to refine our understanding of the dynamic immune landscape within healing defects, moving beyond simplified M1/M2 macrophage paradigms toward metabolically and functionally defined immune subsets. Such high-resolution immune mapping will enable rational design of NP systems that selectively regulate defined immune circuits rather than broadly suppress inflammatory pathways. These advances will generate increasingly complex and multidimensional datasets that necessitate the integration of computational approaches with experimental biomaterials research. In parallel, the integration of systems immunology with materials engineering is likely to reshape NP design strategies. Data-driven modeling approaches incorporating transcriptomic, cytokine, and temporal healing datasets may facilitate predictive optimization of release kinetics, dosing windows, and combinatorial signaling cues. Despite this potential, the implementation of predictive computational frameworks remains constrained by the limited availability of standardized, multimodal datasets integrating material characteristics with immune and regenerative outcomes. The absence of harmonized reporting standards for nanoparticle physicochemical properties, immune endpoints, and longitudinal healing data currently restricts the development of robust and generalizable predictive models. Instead of empirically tuning material parameters, future platforms may be engineered using computational frameworks that anticipate immune–osteogenic interactions across different stages of repair. Advances in responsive material design are also expected to produce adaptive NP systems capable of real-time interaction with evolving microenvironmental conditions. Multi-trigger responsiveness—integrating pH, redox status, enzymatic activity, and mechanical stress—may allow stage-specific immune regulation synchronized with angiogenesis and osteogenesis. Such self-regulating systems could mitigate the risks associated with premature inflammation suppression or prolonged immunomodulation, thereby preserving physiological immune surveillance while enhancing regenerative efficiency. Despite these advances, the long-term consequences of sustained nanoparticle exposure and repeated immune modulation remain insufficiently understood. Questions related to chronic biodistribution, nanoparticle persistence, off-target immune effects, and potential interference with physiological host-defense mechanisms require systematic investigation before widespread clinical implementation can be considered. The incorporation of NPs into advanced composite constructs will likely expand toward spatially graded and temporally dynamic architectures. Emerging fabrication technologies, including multi-material 3D printing and 4D biomaterials, may allow regional control of immune cues within complex defect geometries. By spatially segregating early inflammatory modulation from later osteogenic stimulation, these constructs could more closely replicate native healing cascades. Nevertheless, increasing architectural and functional complexity may introduce additional challenges related to manufacturing reproducibility, batch-to-batch consistency, quality control, and cost-effective scale-up. Establishing reliable fabrication workflows that preserve spatial precision while ensuring regulatory compliance remains a critical barrier to clinical translation. Overcoming these manufacturing constraints may facilitate the development of multifunctional constructs with enhanced biomechanical performance. Furthermore, coupling NP-mediated signaling with mechanically adaptive scaffolds may improve performance in load-bearing defects where structural competence is critical. Translation to clinical application will depend on validation in large-animal and humanized immune models that more accurately recapitulate human bone biology and inflammatory kinetics. At present, the translational value of many preclinical studies is limited by the extensive use of small-animal models that inadequately reproduce the complexity of the human immune system, bone remodeling dynamics, and defect biomechanics. Furthermore, inconsistencies in experimental design, follow-up duration, and evaluation criteria complicate the comparison of therapeutic outcomes across studies and hinder regulatory assessment. Standardization of immune-specific endpoints, long-term remodeling metrics, and mechanical performance assessments will be necessary to establish reproducible benchmarks across studies. Equally important is the development of scalable, reproducible manufacturing pipelines capable of maintaining physicochemical consistency during NP production and scaffold integration. Regulatory pathways for multifunctional composite systems remain complex, and early integration of regulatory science considerations into material design will facilitate eventual clinical approval. Future progress will therefore require interdisciplinary efforts to establish standardized evaluation frameworks encompassing immune-specific biomarkers, biodistribution profiles, long-term safety assessments, and clinically relevant functional endpoints. Such consensus-driven approaches will be essential for transforming promising laboratory concepts into reproducible and clinically deployable osteoimmunomodulatory therapies. Ultimately, the future of NP-based bone regeneration lies in precision osteoimmunomodulation—where biomaterials are designed not merely to deliver therapeutic agents but to dynamically coordinate immune behavior with tissue regeneration. By integrating advances in immunology, materials science, computational modeling, and translational engineering, next-generation NP systems may evolve into intelligent regenerative platforms capable of guiding patient-specific skeletal repair.

## 8. Conclusions

The growing understanding of osteoimmunology has fundamentally reshaped current perspectives on bone regeneration, highlighting the immune microenvironment as a critical determinant of healing outcomes rather than a passive participant in tissue repair. In this context, NPs have emerged as versatile platforms capable of actively regulating immune responses while simultaneously supporting osteogenesis, angiogenesis, and tissue remodeling. Through their tunable physicochemical properties, controlled cargo delivery capabilities, and adaptable surface engineering strategies, NP-based systems can modulate inflammatory signaling, influence immune-cell behavior, and promote the transition from pro-inflammatory to pro-regenerative healing phases.

As discussed throughout this review, inorganic, organic, and hybrid NPs each provide distinct advantages for osteoimmunomodulation. Inorganic systems contribute bioactive ion release and antioxidant activity, organic NPs enable controlled therapeutic delivery and immune targeting, whereas hybrid platforms integrate these complementary functions into multifunctional regenerative systems. Increasing evidence demonstrates that NP-mediated regulation of macrophage polarization, cytokine networks, oxidative stress, and cell-specific signaling pathways can significantly improve bone regeneration and vascular integration in challenging regenerative environments.

Despite these promising advances, several barriers continue to limit clinical translation, including incomplete understanding of long-term immune interactions, variability between preclinical models and human physiology, manufacturing and scalability challenges, and regulatory considerations regarding safety and reproducibility. Addressing these limitations will require interdisciplinary collaboration among materials scientists, immunologists, engineers, and clinicians, together with the development of standardized evaluation frameworks and clinically relevant validation models. Importantly, several knowledge gaps remain unresolved. Current evidence is frequently derived from small-animal studies employing heterogeneous defect models, variable immune profiling methodologies, and non-standardized outcome measures, limiting direct comparison across studies and reducing translational predictability. Furthermore, the widespread reliance on simplified immune classifications, particularly binary macrophage polarization models, does not adequately capture the complexity and temporal dynamics of the osteoimmune microenvironment during bone healing. Resolving these knowledge gaps will require high-resolution analytical approaches, including single-cell and spatial multi-omics technologies, to establish a more comprehensive understanding of immune–bone interactions.

Overall, NP-enabled osteoimmunomodulation represents a promising paradigm for next-generation bone regenerative therapies. Future advances will depend on the integration of mechanistic immunology, adaptive biomaterials, computational modeling, and scalable manufacturing strategies to enable the development of clinically viable osteoimmunomodulatory platforms. Rather than functioning solely as passive delivery vehicles, next-generation nanoparticles are expected to serve as dynamic regulators capable of coordinating immune responses with angiogenic and osteogenic processes in a spatially and temporally controlled manner. Establishing consensus-driven standards for nanoparticle characterization, immune evaluation, long-term safety assessment, and regulatory approval will be essential for translating these technologies from experimental systems to routine clinical practice. By integrating immune regulation with biomaterial design, next-generation NP platforms may enable more precise, personalized, and biologically coordinated approaches to bone regeneration, ultimately transforming skeletal repair from a structurally focused intervention into an intelligent, immune-guided regenerative strategy.

## Data Availability

Data sharing is not applicable to this article, as no datasets were generated or analyzed during the current study.
